# Nano-Silica Carriers Coated by Chloramphenicol: Synthesis, Characterization, and Grinding Trial as a Way to Improve the Release Profile

**DOI:** 10.3390/ph15060703

**Published:** 2022-06-02

**Authors:** Radosław Balwierz, Dawid Bursy, Paweł Biernat, Nataliia Hudz, Mariia Shanaida, Łukasz Krzemiński, Paweł Skóra, Monika Biernat, Wioletta Ochędzan Siodłak

**Affiliations:** 1 Faculty of Chemistry, University of Opole, 45-052 Opole, Poland; natali_gudz@ukr.net (N.H.); wsiodlak@uni.opole.pl (W.O.S.); 2 Department of Drug Forms Technology, Faculty of Pharmacy, Wroclaw Medical University, 50-556 Wroclaw, Poland; dawid.bursy@umw.edu.pl (D.B.); pawel.biernat@umw.edu.pl (P.B.); 3 Department of Drug Technology and Biopharmaceutics, Danylo Halytsky Lviv National Medical University, 79010 Lviv, Ukraine; 4 Department of Pharmacognosy and Medical Botany, I. Horbachevsky Ternopil National Medical University, 46001 Ternopil, Ukraine; shanayda@tdmu.edu.ua; 5 Nanotechnology and Materials Technology Scientific and Didactic Laboratory, Faculty of Mechanical Engineering, Silesian University of Technology, 44-100 Gliwice, Poland; lukasz.krzeminski@polsl.pl; 6 Department of Inorganic Chemistry, Analytical Chemistry and Electrochemistry, Faculty of Chemistry, Silesian University of Technology, 44-100 Gliwice, Poland; pawel.skora@polsl.pl; 7 Department of Haematology, Blood Neoplasms and Bone Marrow Transplantation, Wroclaw Medical University, 50-367 Wroclaw, Poland; mobiernat@gmail.com

**Keywords:** silica carrier, release profile, dissolution study, carbopol, chloramphenicol nanoparticles, drug delivery, sol, Stöber method

## Abstract

Silica nanoparticles were applied as the carrier of chloramphenicol (2,2-dichloro-*N*-[(1*R*,2*R*)-1,3-dihydroxy-1-(4-nitrophenyl)propan-2-yl]acetamide), and were loaded in a 1% carbopol-based gel (poly(acrylic acid)), which allowed obtainment of an upgraded drug form. The samples of silica materials were obtained by means of modified Stöber synthesis, and their morphological properties were analyzed using Fourier transform infrared spectroscopy (FTIR), Brunauer–Emmett–Teller (BET) method, elemental analysis (EA), thermogravimetric analysis (TGA), analysis of the specific surface properties, X-ray diffraction study (XRD), scanning electron microscope (SEM), and dynamic light scattering (DLS) methods, which permitted the selection of the drug carrier. The two obtained silica carriers were coated with chloramphenicol and loaded into 1% carbopol gel. The release studies were then performed. The release results were evaluated using mathematical models as well as model-independent analysis. It was found that the modification of the synthesis of the silica by the sol-gel method to form a product coated with chloramphenicol and further grinding of the silica material influenced the release of the active substance, thus allowing the modification of its pharmaceutical availability. The change in the parameters of silica synthesis influenced the structure and morphological properties of the obtained silica carrier. The grinding process determined the way of adsorption of the active substance on its surface. The studies showed that the proper choice of silica carrier has a considerable effect on the release profile of the prepared hydrogel formulations.

## 1. Introduction

The concept that a specific drug form influences the therapeutical effect is one of the fundamentals of modern pharmacy. An implementation method for this idea is to use a drug adsorbed on a solid carrier. This provides the opportunity to reduce the dose of the used drug while minimizing possible side effects. Moreover, it allows the modification of the active substance release profile as well as improves (or resolves the problem of) the drug solubility. The drugs adsorbed on a solid carrier are successfully applied, for example, in oncological therapies, especially due to the possibility of side effect reduction [1,2].

Various materials can be used as carriers, both inorganic, such as metals (silver, copper, or aluminum) or oxides (oxides of silicon, zinc, titanium, iron, copper, or aluminum), and organic—substances such as polymers, carbon, or biomolecules. The fact that not only the chemical composition of the carriers can be adjusted to the specific drug, but also their surface can be further modified, for example, through a linkage of various active substances, acts as a clear advantage [3].

Silica particles are especially popular. This type of material is characterized by structural and morphological diversity; therefore, it is frequently used in nanotechnology. Silica is known for its thermostability, lack of thermal and electrical conductivity, absence of absorption in the range of 300–800 nm, and chemical inactivity [4]. Silica in the form of nanoparticles, used as the drug carrier, allows for full preparation of a drug form that facilitates the release of the active substance, increasing its bioavailability while modifying the release profile [5,6]. Silica carriers have been implemented in antibiotic therapy. This is mainly due to their high biocompatibility, the ability to control the synthesis process (e.g., controlling the pore size and the specific surface area), and high chemical stability. Furthermore, the advantages include good bioavailability and significant biocompatibility, both in in vitro and in vivo tests. The particle size range of 100–200 nm allows for a prolonged half-life of particles in the blood (no uptake by the liver and filtration in the spleen). On the other hand, nanoparticles in the range of 100–130 nm are also desirable due to the possibility of accumulation in neoplastic tissues [1,7].

In order to synthesize the silica nanocarriers, the Stöber method can be utilized. The nanoparticle formation reaction occurs in a water-alcohol solvent system, with the addition of ammonia to adjust the pH, and a silica precursor [8], most commonly tetraethoxyorthosilane (TEOS) or tetramethoxyorthosilane (TMOS). During hydrolysis and polycondensation reactions, the polymer is formed [9,10], containing nanoparticles with sizes ranging from 5 to 2000 nm [8,11]. The general scheme of the hydrolysis and condensation reactions is as follows:

*Hydrolysis*

≡ Si-O-C_2_H_5_ + H_2_O ↔ ≡ Si-OH + C_2_H_5_OH 

*Condensation*

≡ Si-O-C_2_H_5_ + HO-Si ≡ ↔ ≡ Si-O-Si ≡ + C_2_H_5_OH 

≡ Si-OH + HO-Si ≡ ↔ ≡ Si-O-Si ≡ + H_2_O

The main advantage of this synthesis method is the ability to control the average molecular mass and dispersion of the product. Thus, by changing the conditions of synthesis, it is possible to influence the parameters of the final product.

Silica nanoparticles used for pharmaceutical and medical purposes are characterized by a porous structure that can be used as a potential reservoir and/or carrier for many drug active substances and API [12]. Due to this, for example, nanoparticles with telmisartan (as a drug substance that is difficult to dissolve in water) were obtained with a controllable process of active substance release and an attachment capacity of about 60% of the nanoparticle weight [13]. Nanosilica is also used as a carrier for anticancer drugs, such as doxorubicin [14,15].

The surface of silica nanoparticles can be modified by using the co-condensation, grafting, or imprint coating methods. In pharmaceutical practice, two main strategies for creating combinations of silica nanoparticles with drugs are used: physical adsorption and solvent evaporation [16,17,18,19]. The physical adsorption method was used to attach polymyxin B and vancomycin to the silica nanoparticles. As a result, antibiotic-coated particles with stronger antibacterial activity against *E. coli, P. aeruginosa,* and *S. aureus* were obtained compared to the crystalline form of these antibiotics used at the same concentrations. The produced effect was so significant that the mechanism of action of active substances not using a carrier was described as bacteriostatic, and the antibiotics analogs adsorbed in the pores of nanoparticles as bactericidal [20]. Moreover, rifampicin at the concentration of 0.5 μg/mL did not reveal an antibacterial activity, which may be due to its poor migration through cell membranes. At the same concentration, rifampicin supported by nanoparticles did show antibacterial activity [21].

Chloramphenicol is an antibiotic that is currently obtained synthetically and is classified as a bacteriostatic antibiotic [21,22,23]. Its inhibition of bacterial growth is reliant on the blocking of protein synthesis (it connects to the 50S subunit of the ribosome). The most advantageous feature of chloramphenicol is the broad antimicrobial spectrum, including Gram-positive and Gram-negative bacteria, atypical bacteria such as *Rickettsiae*, and some viruses [24]. Chloramphenicol is indicated for oral administration in the treatment of severe infections such as cholera, meningitis, typhoid, and rickettsia [24]. Unfortunately, in human therapy, it is burdened with side effects, such as aplastic anemia, leukemia, and, when used in children, the so-called gray child syndrome. Due to the high toxicity of chloramphenicol, its concentration for oral administration was determined to be 0.3 µg/kg [25]. For this reason, chloramphenicol is often used externally to treat burns and skin infections. Developing a suitable drug form is crucial as the active ingredient should reach a therapeutic concentration at the site of infection but not penetrate the patient’s bloodstream [26].

The use of silica nanoparticles as a carrier of chloramphenicol seems to be suitable for a modern form of a topical drug product, which, concerning the published results obtained for other antibiotics, suggests the possibility of decreasing the drug concentration thus minimizing side effects and rationalizing the therapy. The use of nano-silica can improve not only the safety profile of chloramphenicol, but also allow to obtain a design-release drug form, which, when compared to a commercially available product, will be characterized by a significantly better antimicrobial activity.

It is significantly essential to find alternative forms for old drugs and attempt to improve their activity. It can be useful in dermatological therapy and may help to reduce antibiotic resistance. Our study aimed to design and develop a silica nanocarrier to form a semi-solid drug form with chloramphenicol and demonstrate its effectiveness in terms of optimizing the release profile of the active substance. The study provides an opportunity to create a new drug form that can be used in further studies. A silica carrier coated with chloramphenicol provides means to obtain a new form of an old drug, which presented a better dissolution study than the reference gel. This enables the possibility of creating a unique formulation containing an active substance, perhaps with better activity against bacteria.

## 2. Results and Discussion

### 2.1. Synthesis and Characterization of Silica Carriers

The reaction of synthesis of silica nanoparticles was carried out in a water-ethanol solution with the addition of ammonia, which increased the condensation of silica precursor, tetraethyl orthosilicate (TEOS) [8]. At the hydrolysis reaction step, the hydroxyl groups (-OH) of the carrier were substituted by alkoxy groups (RO-), which subsequently took part in the formation of siloxane (Si-O-Si) bonds during condensation. As a result, this gradual condensation type polymerization led to a polymer with alternate silicon and oxygen atoms in the main chain.

Three silica nanoparticles (A–C) were synthesized by Stöber’s method using the semi-batch technique. Each of the carriers was synthesized by changing the reaction conditions: temperature, the volume of reagents (ammonia solution, water, ethanol, TEOS), as well as the speed of TEOS addition (the synthesis 1–3, Table 1). In addition, each of the obtained silica particles (A–C) was subjected to grinding, which resulted in the addition of three new carriers, denoted as D–F, respectively.

The obtained nanocarriers were analyzed using the following methods: Brunauer–Emmett–Teller (BET) analysis of nitrogen adsorption–desorption, dynamic light scattering (DLS), thermogravimetry (TG), scanning electron microscope (SEM), X-ray diffraction analysis (XRD), and elemental analysis. The parameters were characterized as specific surface area, pore size, pore-volume, particle diameter, size distribution, thermal stability, and morphology.

The results of the BET analysis are presented in Table 2. It was observed that the specific surface area was different for the carriers A-C. The smallest surface area was found for carrier A obtained according to procedure 1 at a temperature of 65 °C. In contrast, carrier C, obtained by means of procedure 3 at a temperature of 32 °C, demonstrated the highest surface value. The carrier C also showed the highest total pore volume and average pore diameter as determined by the Barrett–Joyner–Halenda (BJH) method. Based on the results of nitrogen adsorption–desorption from the surface of the studied silica, it was shown that the carriers A–C are mesoporous materials with low micropore content. The carriers D–F obtained in the grinding process showed a much higher specific surface than their corresponding A–C precursors. For carrier D, obtained by grinding carrier A, a considerable increase in the specific surface from about 0.2 to 35 m^2^/g was observed. The carriers E and F had a similar surface area, approximately 32 m^2^/g. Grinding carriers also increased the volume and diameter of pores. The most prominent effect was observed for the carrier D. In general, the nitrogen adsorption–desorption isotherms (Figures S1–S6, Supplementary Materials) for each of the studied silica had a similar shape. The analysis of the BJH distribution showed that the carriers D–F, similarly to the A–C analogs, are mesoporous silica with a small proportion of micropores, which predominantly constitute the development of specific surfaces.

The measurement of nanoparticle size of the carriers A–C and their ground analogs D–F was performed using the dynamic light scattering (DLS) method. The statistical summary is presented in Table 3 in the form of histograms (Figures S8–S13, Tables S1 and S2, Supplementary Materials) with a drawn line showing the normal distribution of nanoparticle diameter.

The average diameter of the unground silica nanoparticles A-C, obtained as the result of syntheses 1–3, ranged from 107.7 ± 22.0 to 181.4 ± 16.0 nm. The variation coefficient significantly differed, depending on the synthesis method used, and ranged from 8.8 to 20.4%. The grinding of silica considerably increased the average particle diameter (from 215.3 to 389.7 nm), and the most significant increase was observed for the silica obtained according to synthesis 1. The variation coefficient also increased (from 10.8 to 35.7%) as well as the standard deviation, which, again, was the highest for the carrier D. This may indicate the agglomeration of silica material consisted of particles of various sizes.

The X-ray diffraction analysis (XRD) was performed for the carrier A sample (Figure S16). The wide peak at 2θ = 22° confirmed the amorphous character of unground silica synthesized according to reference [27].

**Figure 1:**
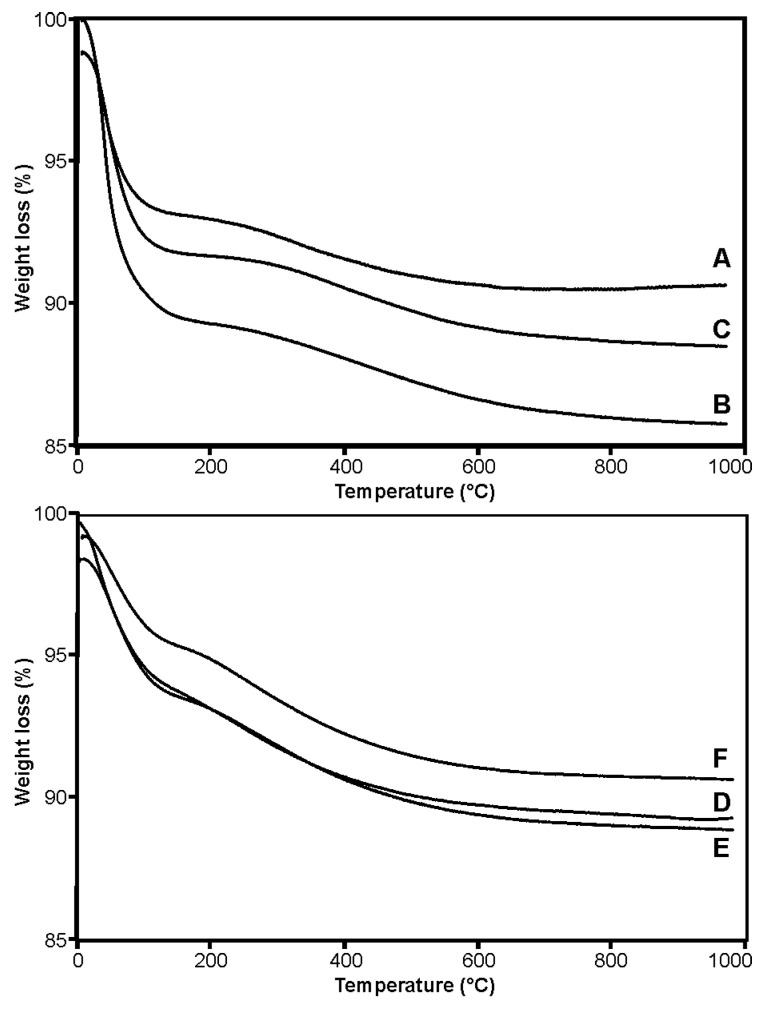
The thermogravimetric analysis (TG) of the silica carriers (A–F).

The thermogravimetric analysis (TG) of the carriers A–F is presented in Figure 1, Figures S18–S23 in the Supplementary Materials. Two stages of weight loss were observed for all of the tested samples. The first step, up to 200 °C, can be attributed to the removal of alcohol and water molecules adsorbed to the silica surface as well as volatile products resulting from the condensation reaction. The loss of mass in this step is related to the endothermic transformation. The second stage, with a slow and relatively steady rate of weight loss, is most likely caused by the removal of ethoxy groups from the silica matrix as well as silanol groups [28]. In the case of carriers A and C, the first loss stage accounts for approximately 7% of the mass. The most significant weight loss (over 10%) in the first stage was found for the carrier B, obtained at 25 °C with an increased amount of TEOS. This may suggest incomplete hydrolysis of the silica precursor—TEOS alkoxide and the distribution of alkoxide groups. For the silica B and C, the second stage indicates a comparable sample weight loss, 3.6 and 3.2%, respectively. This proves a similar structure of the silica matrix obtained as a result of the condensation polymerization (polycondensation) of siloxane. The smallest weight loss (~2.5%) was found for carrier A. This suggests that the degree of polymerization, which strictly depends on the amount and degree of conversion of functional groups (-OH), is slightly lower. The higher temperature (65 °C) used during the synthesis may be the reason.

**Figure 2:**
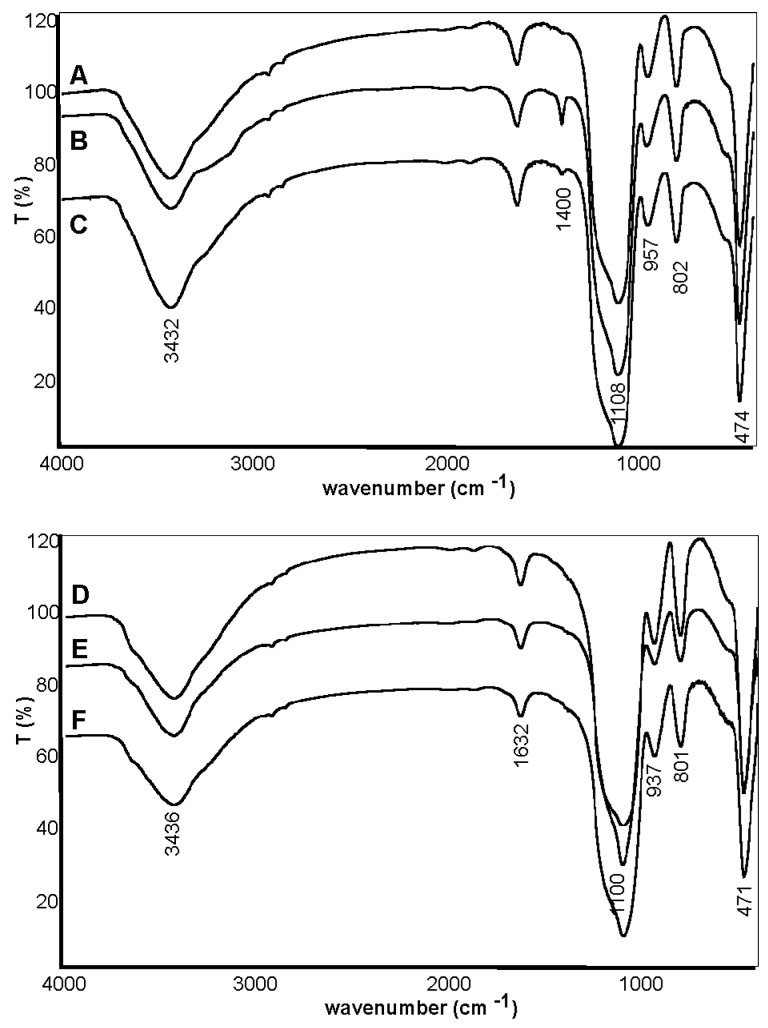
The FT-IR spectra of the studied silica materials A–F.

The FTIR measurements made for the silica materials A–F are shown in Figure 2. A large peak, expanded from 3000 to 4000 cm^−1^, corresponds to the stretching vibrations of SiO-H and HO-H. The bands corresponding to symmetric vibrations of Si-O-Si are present in the range of 1200–1147 cm^−1^ (maximum at 1108 cm^−1^), and the asymmetric ones at 802 and 474 cm^−1^. The bands at 1630 and 957 cm^−1^ correspond to the HO-H bending vibrations [29,30]. Grinding the carrier under normal conditions increased the amount of water on its surface. As a result of the association of adsorbed H-O-H molecules with the Si-OH group, a shift towards lower wavenumbers (from 957 to 937 cm^−1^) is observed. In addition, in the case of the silica synthesized with the increased amount of TEOS, a band at 1400 cm^−1^ was observed, most likely originating from the swing vibrations of the O-H or C-H groups (CH_2_ with TEOS) [31,32].

The elemental analysis was also determined for all of the silica samples (A–F). In the samples A–C synthesized according to procedures 1–3, respectively, trace amounts of nitrogen (0.16, 0.57, and 0.18 wt.%, respectively) were estimated, which indicated the remaining ammonia in the carriers. In contrast, the samples D–F did not show nitrogen, indicating that the grinding process removed the ammonia.

**Figure 3:**
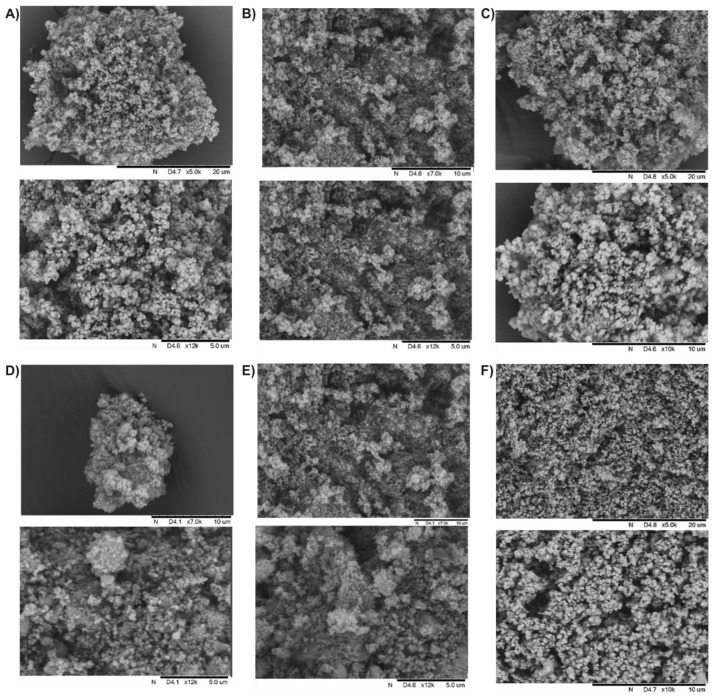
SEM images of the studied silica materials (**A**–**F**).

The scanning electron microscope (SEM) images show a considerable influence of the method synthesis on the morphology of silica materials (Figure 3). The presented samples showed irregular and very porous structures. The tendency of silica nanoparticles to aggregate can be seen, especially for the materials D–F obtained by grinding. The SEM images of silica nanoparticles A–C indicate their spherical shape. Grinding leads to partial destruction of spherical particles and increases the porosity of the surface (specific surface area and pore size).

### 2.2. Synthesis and Characterization of Silicon Nanoparticles Coated with Chloramphenicol

To study the silica carrier coated with the drug substance (chloramphenicol), silica A obtained via synthesis 1 and its ground analog D were chosen. The selected silica A and D were characterized by a diversified specific surface, size and pore volume, and particle size. This should influence the effectiveness of the drug immobilization and its release profile. Chloramphenicol was used as a model drug substance for coating the selected nano-silica materials. The anchorage of the drug on the silica carrier was carried out by dissolving the drug in ethanol and then rapidly evaporating the solvent. In the used method, part of the drug molecules is bonded by physical adsorption, and the rest crystallizes on the surface of silica nanoparticles [16]. In the structure of chloramphenicol, five types of functional groups can be distinguished: two hydroxyl groups, an amide group, two geminal chlorine atoms, a nitro group, and an aromatic benzene ring. The considerable number of polar functional groups on the relatively small carbon skeleton indicates the high polarity of the compound. The presence of amide groups and hydroxyl groups determine the nature of the intermolecular interactions, where hydrogen bonding is the basic interaction type. This is confirmed by the nature of the surface of silica carriers, where hydroxyl groups are predominant. Thus, the physical adsorption of chloramphenicol on the surface of the silica support will generally proceed through the formation of hydrogen bonds in which, more importantly, both the drug substance and the surface of the carrier can act as donors and acceptors of the hydrogen bonds. These strong intermolecular interactions will contribute to the significant ordering of the drug molecules on the surface of silica.

**Figure 4:**
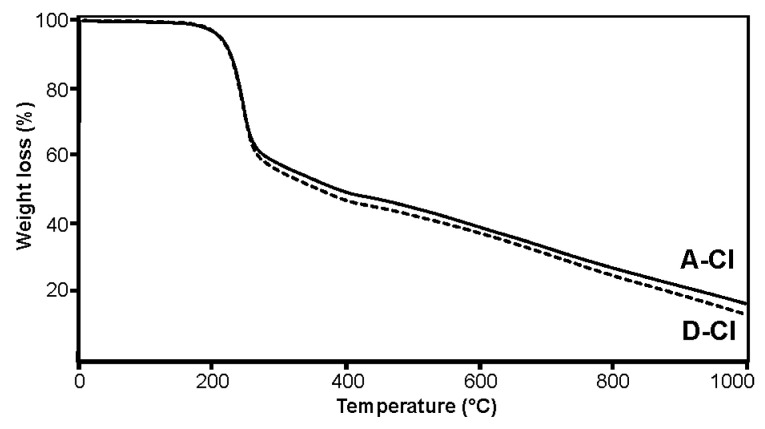
The thermogravimetric analysis (TG) of the materials A_ClPh and D_ClPh coated with chloramphenicol.

The thermogravimetric analysis (TG) confirmed the presence of chloramphenicol on the silica A obtained in synthesis 1 and its, subjected to the grinding process, analog D (Figure 4, Figures S24 and S25). Both analyzed samples reveal similar thermal profiles. Two stages of weight loss were observed on the thermograms of both unground and ground silica coated with chloramphenicol. The first stage, up to 450 °C, constituting, respectively, 52.81 and 55.96% of the mass for the sample silica A and D, corresponds to the decomposition of the organic part connected with the drug. The second step, with the slow rate of weight loss (31.93 and 32.15%, respectively), can be attributed to the gradual degradation of the silica matrix. The obtained results show that the amount of added chloramphenicol used for coating the nanoparticles in both carriers is comparable.

As expected, coating the silica with the drug substance resulted in a reduction of the specific surface area of silica material D by filling its pores. The volume of pores was reduced by more than tenfold (Table 2, item D and D_ClPh, Figure S7). Despite these changes, the silica with the antibiotic retained its amorphous character, as evidenced by the XRD curve (Figure S17).

The anchorage of chloramphenicol on the silica nanoparticles caused an increase in the diameter of silica A particles by approximately 25%, as determined by dynamic light scattering (DLS) (Table 3 and Table S3, Figures S14 and S15). In contrast, coating the silica D sample resulted in a decrease in the particle size. The possible reasoning behind this phenomenon includes a breakdown of aggregates as a result of the subsequent grinding of silica material during the immobilization of the drug substances. The variation coefficient was 12% and 20.7%, while the standard deviation was 21.3 and 32.3, respectively, for materials A_ClPh and D_ClPh. The D_ClPh particle size was slightly smaller than that of A_ClPh.

**Figure 5:**
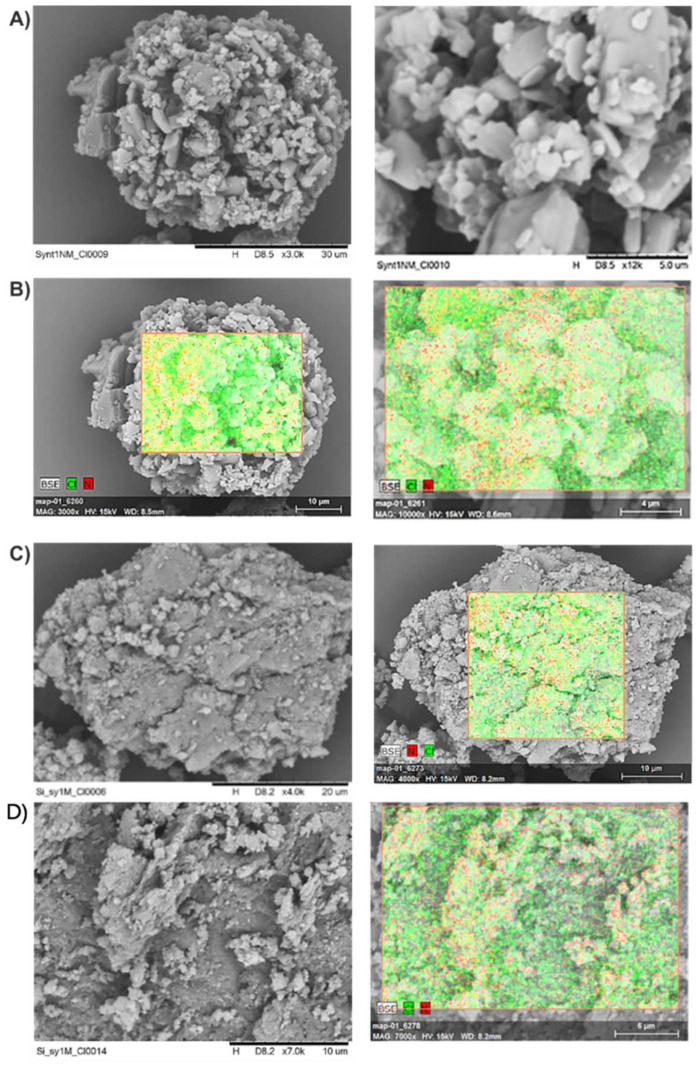
SEM images of the carriers A and D coated with chloramphenicol: (**A**) unground, coated carrier; (**B**) EDS mapping of unground, coated (A_ClPh); (**C**) ground, coated carrier; and (**D**) EDS mapping of ground, coated (D_ClPh).

The SEM images of the carriers A and D coated with chloramphenicol are presented in Figure 5. As is evidenced, the silica (A) particle coated with the drug substance remained spherical (A_ClPh). The surface of the particle is porous, and the observed pores are deep. The elemental mapping of the chemical composition, showing the content of chlorine and nitrogen atoms, was made using the EDS method and confirmed the presence of chloramphenicol on the surface of the particle. It is also worth noting that the greatest amount of the drug substance is located in the pores of silica, although some amount in the crystal form is also present on the surface. Double-ground silica carrier particles (D_ClPh) (before coating with the drug and during coating) were heavily damaged, which caused diverse morphology. Some of the particles remained spherical, but most of them broke, making their surface much smoother, without deep or wide pores. This caused the drug substance to be evenly distributed over the surface of the particles and not accumulated in the pores.

The physical properties, such as the density and viscosity of ointment preparations, have a significant impact on the profile and rate of release of the active substance. The rheological tests of 1% carbopol gel were carried out, using both the silica-coated with chloramphenicol and chloramphenicol alone in the crystalline form (without silica). To control the content of chloramphenicol in the prepared gels, high-performance liquid chromatography (HPLC) in the reversed-phase system was used. In the experimental conditions, the average retention time of chloramphenicol was 3.28 min. The lack of signals from other substances, apart from spectral disturbances near the front of the chromatogram, resulted from the use of other solvents in the mobile phase (acetonitrile and deionized water) and other solvents used for the extraction of chloramphenicol from the gel (ethanol), indicated that the samples were properly prepared.

The average peak area of two injections was used to calculate, using the standard curve, the concentration of chloramphenicol in the test sample, and, subsequently, these data were converted to the content of chloramphenicol in the entire volume of the gel. The expected content was obtained (Table 4).

In order to compare the rheological properties of the prepared gels and to determine the effect of silica particles and the addition of chloramphenicol (0.5% by weight), the apparent viscosity was measured for: the carbopol-based gel without chloramphenicol and silica, the gel with chloramphenicol, and the gel with the drug adsorbed on silica A as well as on the ground silica D (Table 5). The 1% carbopol gel used demonstrated thixotropic properties, including low fluidity at rest, easy spreadability during the application, and the ability to create a stable film at the site of gel application [33]. The viscosity of the 1% carbopol gel without the addition of silica particles coated with chloramphenicol increased non-linearly with the increase of shear time; this relationship is typical for an expansion fluid [34]. The apparent gel viscosity was determined to be 272.71 [Pa·s], which is consistent with the literature [33]. The viscosity of the gel with a 0.5 wt.% addition of the crystalline chloramphenicol significantly decreased to the value of 248.85 [Pa·s]. The silica-coated with the antibiotic in the amount of 0.5 wt.%, regardless of the degree of its grinding, caused an increase in viscosity of the gel when compared to the viscosity of the gel only with chloramphenicol. Thus, the addition of silica nanoparticles counteracts the observed decrease in viscosity. It is worth noting that the carbopol gel based on the unground silica coated with the drug demonstrated slightly lower viscosity than the 1 wt.% gel without additives (a decrease of approximately 10%). The grinding of the silica increased the contact surface with the gel, which in turn increased the order of gel molecules around the nanoparticles silica-coated with the antibiotic, which increased the viscosity of the system. The size of silica particles with the antibiotic and their ability to aggregate is also important.

### 2.3. Study of the Release of Chloramphenicol from the Carbopol-Based Gels

The release of the active substance from gels containing nanoparticles took place at a faster rate when compared to gels without the nanoparticles. As expected, the particles with the smallest diameter contained a larger amount of the active substance on their surface and, thus, released a higher portion of the drug faster. On the other hand, the largest particles released the active substance at a slower rate. The obtained results led to a conclusion that the diameter of the nanoparticles affects the amount of the released active substance.

**Figure 6:**
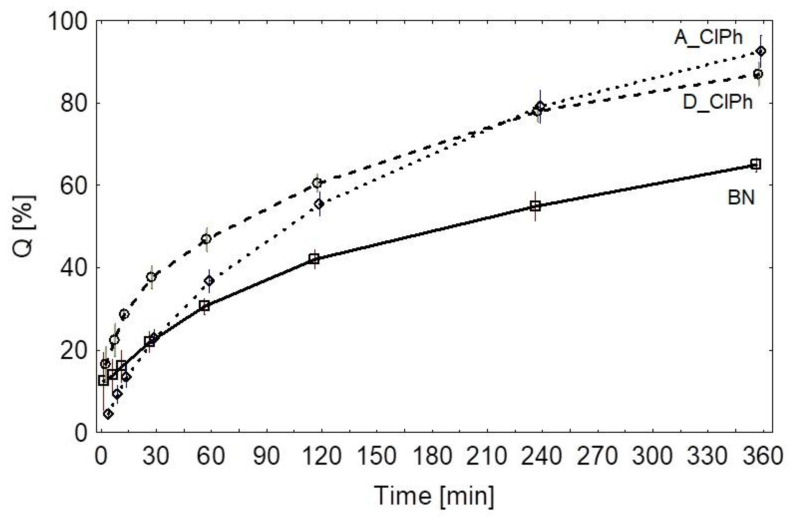
Q diagram for the values of the released percentage over time. Mean; Whisker: Mean ± 0.95 Conf. Interval. **○** mean release points of D_ClPh; **□** mean release points of BN; **◊** mean release points of A_ClPh.

This is also confirmed by the kinetics of the release presented in Figure 6. The formulation with ground silica carrier coated with chloramphenicol released the active substance faster. The rate of release, especially the initial release, may depend on the presence of non-adhered chloramphenicol in the formulation or only adsorbed to the surface, which releases almost immediately. The larger the molecule and the more porous the texture, the potentially more difficult it is for chloramphenicol to be eluted by the acceptor fluid. This is reflected in the comparative results for the A_ClPh and D_ClPh formulations. The difference in the presented release profiles may also result from the faster release of less porous silica particles (D_ClPh) versus more porous particles (A_ClPh). This is due to the difference in the amount of the drug substance on the surface, as well as in the deeper layers of silica carrier in these cases. The more spherical nature of the carrier in the D_ClPh gel resulted in better contact between the acceptor fluid and the carrier and, thus, resulted in a more efficient elution of the antibiotic. The particle size also influenced the physical adsorption surface area of chloramphenicol (larger size for the formulation of D_ClPh). Thus, both the adsorption surface area, particle size, and particle morphology affect the release profile of chloramphenicol from the tested gels. The detailed results are presented in the Supplementary Materials (Section S2).

In order to visualize the phenomenon of the chloramphenicol release from the prepared gels, the analysis of release profiles based on the mathematical models, model-independent analysis, and empirical model compliant with EMA and FDA recommendations, i.e., the Weillbuir model, were used. The empirical models were tested with the objective of matching the drug release data, as shown in Table 6, using the best correlation coefficient (r).

The conducted model matching [35] showed that the reference gel (B.N.) can be described using the Higuchi model (R^2^ = 0.997), the gel based on the ground silica carrier, using the Papas model (R^2^ = 0.993), and the gels based on the unground silica carrier, using the Higuchi model (R^2^ = 0.996). Both models appear to be the most optimal for describing release profiles of the prepared gels. The mathematical calculations are presented in the Supplementary Materials (Section S3).

Based on the pharmaceutical availability study, the release efficiency of D.E. was determined (Table 6 and Supplementary Materials Section S3), and the following basic descriptive statistics were calculated: deviation (SD) and standard error (SE), coefficient of variation (CV, RSD) and 95% significance interval for the mean (95% CI) (Table S30). The determined coefficients of variation did not exceed the CV = 10% limit defined by the FDA and EMA guidelines at either time point. In the next step, the statistical significance of differences between the mean values of compared parameters was evaluated using parametric analysis of variance (ANOVA) (*p* < 0.05) (Table S31 and Figure S27).

Chloramphenicol coated nanoparticles have shown higher release efficiency (D.E.) values which were statistically analyzed. Statistically significant differences were found between gels without nanoparticles and gels with nanoparticles (*p* < 0.05) (Table S31). The formulation based on ground silica carrier demonstrated the highest release efficiency. Therefore, this formulation was characterized with the fastest chloramphenicol release rate. The formulation without the nanoparticles was shown to have the lowest release rate. The average dissolution time was consistent with that presented in the graph. Initially, the D_ClPh formulation released the highest amount of the active substance (mean MDT = 90.18 min), followed by BN (reference gel, mean MDT = 107.18 min) and silica based on an unground silica carrier (mean MDT = 117.80 min), visible especially in the initial release stage. Mathematical calculations are presented in the Supplementary Materials (Section S4).

#### 2.3.1. The Weilbull Model

The obtained three release profiles were described using the empirical Weibull model recommended by the FDA and EMA guidelines.

Values of the estimated equations representing the course of the release profile:

$$BN=100\left(1-e^{{\left(-0.00282t\right)}^{0.54563}}\right)$$

$$MK=100\left(1-e^{{\left(-0.00834t\right)}^{0.55761}}\right)$$

$$K=100\left(1-e^{{\left(-0.00455t\right)}^{0.57281}}\right)$$

The values of the estimated parameters fitted to the model are shown in Table 7. Figure 6 shows the function curve and mean values of the percent released from the tested formulations, and Figure S29 shows the graphical match to the model. Statistical testing results for the Weibull model are presented in Table S35.

#### 2.3.2. The Mahalonobis Distance

The Mahalanobis distance is the distance between release profiles that differentiates the respective components’ contributions and exploits the correlations between them. What was established was the mean and the difference between the compared release profiles by means of determining the multivariate Mahalanobis distance (MSD) for each pair (BN vs. A_ClPh and BN vs. D_ClPh) along with the recommended 90% significance interval (Table 8).

Based on the results, it was determined that the value of the upper limit of the confidence interval is higher than the maximum acceptable value. This indicates that the profiles cannot be considered as similar.

#### 2.3.3. Model-Independent Coefficients of Similarity and Difference (F1, F2)

The parameters describing the release of the active substance from the six tested and referenced formulation samples under identical conditions were determined, followed by the calculation of the average percentage of the released active substance from the tested formulation QT_j and the reference formulation QR_j for each time interval (j). The release curves are considered similar if F1 is close to 0 (0–15) and F2 is close to 100 (50–100) (Table S37).

The values of the difference coefficient (F1) assume a value above 15; therefore, statistically significant differences were demonstrated in the release profiles of the gel without silica carrier compared to the other gels containing the carrier. All the compared release profiles assume a probability factor (F2) value below 50, which allowed rejecting the hypothesis of equivalence of all the formulations. Thus, based on the results, it was determined that the described profiles could not be considered equivalent/similar.

In comparison to all the formulations, the release rate from the D_ClPh formulation was the fastest. The analysis of the release profiles shows that the rate of release of chloramphenicol is dependent on the diameter of the coated nanoparticles. It occurred more rapidly for the smaller diameter nanoparticles. The release from particles with a diameter smaller than 250 nm occurred faster than the release of the crystalline (available commercially) form of the drug substance suspended in the gel (BN).

An attempt to compare the release profile of a poorly soluble substance from the surface of silica nanoparticles with the release profile of the crystalline form of the substance showed that it is possible to improve the release efficiency of the active substance from the gel by using silica carriers, which aim is to increase the local availability of poorly soluble substances.

The release of the active substance from the nanoparticles with a diameter below 250 nm is faster than from the standard gel (BN). The use of nanoparticles allows modifying the release of the active substance. In both cases, an effect of accelerated release from the reference gel (BN) was obtained (Figure 6).

The reduction in the diameter of nanoparticles used as carriers of active substances may cause an increase in the specific surface area to which active substances can be attached. Finally, it increases their adsorption properties. The diameter of nanoparticles affects the release rate of the coated active substance. The drug substance is released faster from particles with a smaller diameter.

We presume that the faster release of the active substance means potentially more effective topical therapy. Thus, elaboration of a silica carrier in the dermatological formulation of chloramphenicol is reasonable.

## 3. Materials and Methods

### 3.1. Materials

Tetraethoxysilane—TEOS 99.99% (Aldrich Chemistry, Darmstadt, Germany); water ammonia solution 25% (Chempur, Piekary Slaskie, Poland); anhydrous ethanol (46.07 g/mol, JT Baker, Phillipsburg, NJ, USA); purified water (obtained with Polwater deionizer CNX-100T 717); sodium hydroxide (Chempur, Piekary Slaskie, Poland); chloramphenicol FPV (PPH Galfarm, Cracow, Poland); and carbopol 934P (BF Goodrich, Cleveland, OH, USA).

### 3.2. Characterization Techniques

The estimation of the silica carrier and silica carrier coated with chloramphenicol particle size was performed by Dynamic Light Scattering (DLS) using the Malvern Zetasizer Nano-ZS ZEN 3600 device (Software: v7.13, Worcestershire, UK) in accordance with the references [36,37]. The samples of silica carrier and silica carrier coated with chloramphenicol were prepared by weighing 1 mg of powder crushed in an agate mortar and suspending it in 2 mL of deionized water by shaking in an ultrasonic bath for 5 min to disintegrate the aggregates.

Fourier transform IR (FTIR) analyses were accomplished using a Nicole Nexus 2002 FTIR (Software Omnic 5.2a, ThermoNicoletInc., Madison, WI, USA) spectrometer from 4000 to 400 cm^−1^ with a 2 cm^−1^ resolution. The samples of the silica particles were prepared in the form of tablets made of silica powder and KBr. 

Elemental analysis (EA) was performed using the 2400 Series II CHNS/O System (Perkin Elmer) apparatus (Wellesley, MA, USA). The weight of each sample ranged from 1.5 to 2.5 mg. The analysis for each sample was performed three times. Measurement errors were considered according to the instrument manual: C ± 0.30%; H ± 0.09%; N ± 0.09%.

Thermogravimetric analysis (TGA) data were obtained in the platinum pan (capacity 100 µL) in a dry box. Using the TGA 2050 (TA-Instruments, New Castle, DE, USA), the sample was pierced while being inserted into the instrument’s dry nitrogen atmosphere. The TGA data were collected at 10 °C/min under nitrogen. 

Nitrogen adsorption–desorption isotherms were obtained using a Gemini VII 2390t and VacPrerp 061 apparatuses (software MicroActive 5.0, Micromeritics, Norcross, GA, USA) after degassing the samples under vacuum at 150 °C for 12 h before measurements. The total surface areas were calculated using the BET method, and the average pore diameter was calculated using the BJH method.

Analysis of the specific surface properties was performed by varying the nitrogen pressure (>99.9999%) at 65 points and indirectly by measuring the amount of adsorbate during adsorption and desorption of nitrogen from the surface of the test materials, in the range of 0.00125 to 0.995 p/p^0^ of saturated vapor pressure at 77.35 K. The time of steady-state equilibration of the adsorbate pressure was 10 s. In terms of specific surface area property measurements, results were obtained based on a single-point BET analysis (Brunauer, Emmett, Teller) and multi-point BET analysis in the range of 0.05–0.3 p/p^0^ saturated vapor pressure, pore size distribution by BJH method in terms of adsorption and desorption.

An X-ray diffraction study was performed using a D2 Phaser X-ray diffractometer (Bruker) on silica carrier samples that were micronized in an agate mortar immediately before application onto an aluminum plate. The tested sample was exposed to X-rays generated by a ceramic sheathed Cu lamp powered by a 30 kV, 10 mA generator. The test was performed in the range from 5 to 36° 2Θ with a measurement every 0.02° 2Θ for 1 s.

A scanning electron microscope (SEM) with energy dispersive X-ray spectrometry (EDS) experiment of the silica samples was carried out on a Hitachi model TM 3000 electron microscope (Hitachi High-Technologies Corporation, Tokyo, Japan). The samples were fixed on an aluminum sample stub and coated with palladium by conventional sputtering techniques. The employed accelerating voltage was 5–15 kV for SEM.

Brookfield DV-II rheometer was used to investigate the apparent viscosity of gels. The gel sample (0.5 mL) was placed in a thermostatic holder, and after reaching a temperature of 32 °C the measurement was started at a set shear rate of 0.384 s^−1^. The test was carried out for 4 min—every 10 s, the viscosity was measured automatically, and the last measurement was included in the analysis.

The chloramphenicol content in the carbopol gel was determined by high-performance liquid chromatography (HPLC). The analysis was performed using an Agilent Infinity 1260 HPLC apparatus with a ThermoScientific column, 4.6 × 150 mm, grain size 5 μm, C18 hypersil Gold bed, mobile phase mixture of acetonitrile and water in a volume ratio of 40:60. The time of one measurement was 6 min [38]. The injection volume was 20 μL, and the temperature of the thermostat was 25 ℃. A photodiode array detector with signal analysis at a detector wavelength of 278.4 nm was used [39]. The gel sample (100 mg) was transferred to a 25 mL class A volumetric flask and filled with ethanol. The sample was stirred for 15 min using an ultrasonic cleaner, then placed in the refrigerator for 48 h before measurement. The average retention time was 3.28 min. The selectivity of the method was checked by repeating the analysis with a gel containing silicon nanoparticles uncoated with chloramphenicol. The analysis showed no signals in the R.T. region for chloramphenicol. The precision of the method was confirmed by injecting the standard solution 6 times and obtaining an RSD of 1.95%.

The grinding of the silica material was carried out using a Mono Pulverisette 6 planetary mill (Fritsch) with a single grinding bowl equipped with 30 agate balls.

The release study of chloramphenicol from the silica carrier was performed in a Hanson Research SR8PLUS pharmacopoeial paddle dissolution apparatus with Enhancer cell vessels with an Agilent 850DS autosampler. The obtained chloramphenicol coated silica carriers and the crystalline form of chloramphenicol, respectively, were suspended in a previously prepared 1% carbopol gel. The assay was conducted at 32 °C with a constant stirring speed of 90 rpm for 6 h. The acceptor medium in the study was demineralized water. The membrane separating the donor from the acceptor was the Cuprophane dialysis membrane, with a molecular cut-off ratio of 10,000 Da; the thickness of the non-wetted membrane was 11.5 μm. The study was conducted using two formulations obtained from respective syntheses of chloramphenicol coated nanoparticles synthesis 1 milled (denoted hereafter as D_ClPh) and unmilled (denoted hereafter as A_ClPh) against a reference formulation prepared from chloramphenicol without a carrier (denoted hereafter as B.N.), 6 units for each formulation. The weights of the gels applied to the immersion chambers were verified to calculate the total active substance content for each chamber. At appropriately programmed measurement points, the autosampler sampled 2 mL of solution from each chamber. The study provided for 8 measurement points: (I)—5 min, (II)—10 min, (III)—15 min, (IV)—30 min, (V)—60 min, (VI)—120 min, (VII)—240 min, and (VIII)—360 min. Samples of the acceptor solution collected by autosampler were examined spectrophotometrically at λ = 278 [40].

### 3.3. The Analysis of Release Profiles and Statistical Analysis

The D.E. release efficacy, a parameter independent of the kinetic model, was determined for each sample of the six drug forms representing the three formulations, which were compared on the basis of the pharmaceutical availability studies. Basic descriptive statistics were first calculated for the three mean values of the parameter: standard deviation (SD) and standard error (SE), variation coefficient (CV, RSD), and 95% significance interval for the mean (95% CI). The normality of their distributions was assessed by the Shapiro–Wilk W test. Levene’s test was used to assess the homogeneity of variance. All the analyzed variables met the assumptions of both the normality of distribution and variance homogeneity. In no time point did the coefficients of variation not exceed the CV = 10% limit defined by the FDA and EMA guidelines. In the subsequent stage, the statistical significance of differences between the mean values of compared parameters was assessed using the ANOVA parametric variance analysis. For post hoc multiple comparisons, the Fisher’s NIR test of least significant difference was used. In all applied analyses and statistical tests, the significance level of α = 0.05 was assumed.

The following mathematical matches for comparison of release profile similarity were used:

- model-dependent based on Weibull function;
- model-independent based on Mahalanobis distance;
- model-independent similarity and difference coefficients (F1, F2).

The obtained three release profiles were described using the empirical Weibull model recommended by the FDA and EMA [41,42] guidelines as well as related to mathematical models, i.e., zero-order kinetics, first-order kinetics, Higuchi, Hixson–Crowell, and Pepas models. The estimation of parameters (*k*, *b*) for the Weibull model was performed using the Weibull function form defined according to the formula:

$$Qt=100*(1-Exp\left(-k*t{)}^{b}\right)$$

minimizing the *S* loss function of least squares was defined as:

$$S=\sum {\left(Obs-Pred\right)}^{2}$$

The minimization procedure for the loss function *S* defined as per above was performed based on a nonlinear Quasi-Newton iterative algorithm. The nonlinear estimation procedure was carried out using random initial values of the parameter estimators *k* and *b*. The initial step length in the iterative procedure was set as 0.001 by adopting a convergence criterion of 1 × 10^−6^.

The mean difference between the compared release profiles was calculated by determining the multivariate Mahalanobis distance (MSD) along with the recommended 90% significance interval for each pair (BN vs. A_ClPh and BN vs. D_ClPh).

Parameters describing the release of six samples of the tested and their reference formulations under identical conditions were determined, followed by the calculation of the mean percentage of released active substance from the tested formulation QT_j and the reference formulation QR_j for each time interval (j). The release curves are considered similar if F1 is close to 0 (0–15) and F2 is close to 100 (50–100).

### 3.4. Synthesis Procedures

#### Silica Carrier Synthesis

Three carriers were obtained according to the Stöber method [8] with semi-bath modification according to the parameters presented in Table 1.

Deionized water, ethyl alcohol, and ammonia water solution were added to a thermostated glass reactor in appropriate proportions (Table 1). The reaction mixture was heated to the set temperature (25, 35, and 65 °C) and stirred continuously at 150 rpm. Once the assumed temperature was reached, TEOS was automatically injected at the specified rate. The pH value was controlled using a pH-meter linked to the Tiamo software and maintained above the value of 11 by adding ammonia solution to the reaction mixture in 1 mL doses. The visual appearance of the reaction mixture changed over time. For synthesis 1, opalescence appeared after 4 min, turbidity after 7 min, and sedimentation of a white suspension after 9 min. The total time of synthesis was 60 min. For synthesis 2, opalescence appeared after 3 min, turbidity after 5 min, and sedimentation after 7 min. The synthesis was carried out for 105 min. For synthesis 3, the time of the appearance of opalescence was 8 min, turbidity—12 min, and sedimentation—20 min. The process took 120 min to complete. At the end of the synthesis, the reaction mixture was in the form of a white suspension.

Then, the reaction mixture was allowed to cool for 30 min and was centrifuged for 5 min (800 rpm). The prepared mixture was poured into Petri dishes and left for 48 h for the solvent to evaporate. The applied procedures 1–3 led to obtaining the carriers A–C, respectively.

### 3.5. Silica Carrier Grinding

The obtained nano-silica carriers A–C were vacuum dried at 30 °C (−0.5 bar) for 24 h. Then, 2500 mg of each carrier was ground in an 80 mL grinding bowl along with 30 agate balls of 10 mm diameter each. Three grinding cycles were carried out at 200 rpm for 2 h each. As a result, the carriers D-F were obtained. Grinding was carried out to develop the material’s specific surface.

### 3.6. Coating of Silica Carrier with Chloramphenicol

A solvent evaporation method was used to coat the silica carrier with chloramphenicol [43]. A previously weighed 150 mg of chloramphenicol was placed in an Eppendorf tube. Subsequently, 0.75 mL of 96% ethanol was added and mixed intensively to obtain a clear solution. Then, 150 mg of the dried form of nanoparticles obtained by synthesis 1 was added to such prepared solution and shaken. An ultrasonic bath at a continuous program of 37 Hz for 20 min was used. Subsequently, the prepared aqueous-ethanol suspensions of silica nanoparticles with chloramphenicol were poured into Petri dishes and transferred to a circulating heating oven and dried at 40 °C for 48 h. The resulting powder was then dried again at 60 °C for 15 min. The remaining amount was ground in an agate mortar. This procedure resulted in the preparation of the carrier A_ClPh.

### 3.7. Coating of Ground Silica Carrier with Chloramphenicol

After the grinding phase, 1.500 g of the powdered silica carrier D was removed from the grinding bowl, and a solution of 1.000 g of chloramphenicol in 10 mL of anhydrous ethanol was added. The solution was transferred to a grinding bowl, and three grinding cycles (120 rpm) were carried out for 8 h each. In the next step, the bowl was opened and left in a vacuum dryer at 30 °C, −0.5 bar for 24 h. The reassembled bowl was placed on the dryer, and three grinding cycles were carried out at 120 rpm for 8 h each. Again, the bowl was opened and left in a vacuum dryer at 30 °C, −0.5 bar for 24 h. This procedure resulted in the preparation of the carrier D_ClPh.

### 3.8. Preparation of the Carbopol Gels

To evaluate the suitability of the obtained chloramphenicol coated carriers in dermatological therapy (ground with chloramphenicol (D_ClPh) and unground with chloramphenicol (A_ClPh)), the prepared supported chloramphenicols and crystalline chloramphenicol (BN) were suspended in the carbopol gel at identical concentrations (0.5% *m*/*m*). The gels were prepared using carbopol 934P at a concentration of 1% [44,45], which was mixed with the chloramphenicol coated silica carrier and crosslinked with 10% sodium hydroxide solution. After the gels were prepared, they were allowed to stand for 60 min at room temperature, and then the pH was checked. An Emeltron CPC-511 pH meter with the IJ44C combination electrode from Ionode was used. The pH meter was calibrated using standard solutions of pH 4, 7, and 10. The prepared gels were transferred to a refrigerator for 24 h for degassing and stored at a temperature of 8 °C until further use.

The scheme of the experiment is presented in the Supplementary Materials.

## 4. Conclusions

Changing the parameters of silica carriers’ synthesis in the Strober method allows the modification of the structure and morphological properties of the obtained silicon materials. Additionally, these changes are enhanced by performing a simple technical operation of grinding the obtained silica carrier. The obtained silica particles were used as a carrier for the medicinal substance chloramphenicol. It was shown that the grinding of the carrier determines the way of adsorption of the active substance on its surface. This, in consequence, permits the modification of the release profile of the prepared hydrogel formulations. Significant differences were found between the release profiles of the reference substance from the hydrogel formulation of the drug and the analogous formulation with the carrier coated with the reference substance. The ground silica carrier is shown to have a higher amount of the active substance on the surface and, thus, shows a faster release than the unground silica carrier with chloramphenicol. For dermatological application, a gel with an unground silica carrier with chloramphenicol is a more effective formulation. Its release profile is more predictable and does not rapidly release a portion of the active drug. This profile can be useful in the development of a new form of dermatological products; however, the final formulation requires further research using microorganisms in order to verify the antibacterial activity against the reference bacteria strains. The use of the obtained silica carriers in semi-solid hydrogel drug form increases the pharmaceutical availability of a poorly soluble active substance, chloramphenicol, in comparison to hydrogel with an active substance without the carrier. This provides an opportunity to create a drug form for dermatological use with a higher concentration of the active substance at the site of administration (target effect). The study provides the possibility to continue research to verify the antibacterial activity of the tested formulation. It appears that the silica carrier coated with chloramphenicol inserted into carbopol gel can be more effective than commercially available ointments with this active ingredient, however this suggestion needs to be assessed by means of microbiology studies.

## Figures and Tables

**Table 1:** Parameters of the synthesis of silica nanoparticles.

Parameters		Synthesis 1 Carrier A	Synthesis 2 Carrier B	Synthesis 3 Carrier C
Temperature [°C]		65	25	35
Volume of added reagents [mL]	ammonium hydroxide 25%	4.8	6.0	4.8
	water	4.8	6.0	4.8
	ethanol 96%	72.0	90.0	72.0
	TEOS	6.4	8.0	6.4
TEOS insertion rate [mL/min]		3.2	4.0	3.2
Volume of added ammonia solution [mL]	prior to the application of TEOS	2.4	13.0	0
	after TEOS application	14.5	15.0	13.0

**Table 2:** Results of the nitrogen adsorption–desorption analysis (BET method).

Material	A	B	C	D	E	F	D_ClPh
Specific surface [m^2^/g]	0.1871	13.8976	23.8035	35.0244	32.8918	32.7435	3.3736
One-point analysis of specific surface area (p/p° = 0.25)	0.1571	13.5829	22.9921	34.0508	32.2388	31.9001	3.1355
Total pore volume in the range 1–300 nm (BJH adsorption) [cm^3^/g]	0.000327	0.027005	0.050779	0.106719	0.105568	0.104813	0.010528
Total pore volume in the range 1–300 nm (BJH desorption) [cm^3^/g]	0.000292	0.029091	0.068072	0.134964	0.129747	0.121554	0.011746
Average pore diameter (BJH adsorption) [nm]	5.5172	6.8381	7.3497	10.5674	11.5499	11.2547	8.3944
Average pore diameter (BJH desorption) [nm]	12.9577	7.7064	10.1059	13.6115	13.1257	12.8671	11.7519

Materials A–C obtained using Stöber’s synthesis; materials D–F obtained by means of grinding materials A–C; A_ClPh—formulation based on silica A and chloramphenicol (1:1); D_ClPh—formulation based on ground silica A (carrier D) and chloramphenicol (1:1).

**Table 3:** Statistical summary of the particle size measurements (nm) for silica nanoparticles obtained using the dynamic light scattering (DLS) method.

Material	Average [nm]	Std. Dev.	RSD	Minimum [nm]	Median [nm]	Maximum [nm]
A	107.7	22.0	20.4%	62.8	112.8	131.0
B	181.4	16.0	8.8%	143.8	186.6	196.9
C	125.1	16.0	12.8%	84.2	131.9	137.3
D	389.7	139.3	35.7%	112.2	438.0	556.5
E	215.3	23.3	10.8%	188.8	212.9	289.2
F	260.0	77.8	29.9%	168.7	257.3	440.4
A_ClPh	178.2	22.14	12.0%	141.8	182.2	241.2
D_ClPh	156.3	32.3	20.7%	100.3	151.8	229.1

Materials A–C obtained using Stöber’s synthesis; material D–F obtained by means of grinding materials A–C; A_ClPh—formulation based on carrier A and chloramphen-icol (1:1); D_ClPh—formulation based on carrier D and chloramphenicol (1:1).

**Table 4:** Content of the active substance in a 1% carbpol gel as determined by the HPLC method.

Sample	Weight [mg]	AUC Average	CAP [mg]	Content [mg/100 g]	Content Relative to Expected [%]
Only ClPh 0.5% (BN)	100.36	566.0223	0.3955	492.6371	98.53%
A_ClPh (1:1) 0.5%	103.24	624.4009	0.4368	528.8567	105.77%
D_ClPh (1:1) 0.5%	101.35	582.7027	0.4073	502.3668	100.47%

BN—formulation based only on chloramphenicol (without the silica carriers); A_ClPh—formulation based on carrier A and chloramphenicol (1:1); D_ClPh—formulation based on carrier D and chloramphenicol (1:1); CAP—capacity; AUC—area under the curve.

**Table 5:** Statistical summary of the apparent viscosity measurements and the pH of gels.

Type of Gel	Average [Pa·s]	S.D. [Pa·s]	Coefficient of Variation [%]	pH
Without the silica and chloramphenicol	272.71	20.45	7.50	7.02
Without the silica and containing only chloramphenicol BN	248.85	0.79	1.62	6.91
Silica and chloramphenicol A_ClPh	262.01	6.47	2.47	6.78
Silica ground with chloramphenicol D_ClPh	361.77	27.52	7.61	6.89

Chloramphenicol concentration = 0.5 [wt.%].

**Table 6:** In vitro release kinetic models of silica carriers.

Analysis Based on Mathematical Models [R^2^]						Model Independent Analysis	
Formulation	First-Order Kinetics	0-Order Kinetics	Higuchi	Hixson–Crowell	Pepas	Release Efficiency D.E.	Mean Dissolution Time MDT [min]
BN	0.98274	0.93977	0.9968	0.97108	0.98975	0.45618	107.18720
D_ClPh	0.99026	0.89692	0.98464	0.96958	0.99338	0.65125	90.186017
A_ClPh	0.99600	0.93182	0.99622	0.99437	0.98206	0.62205	117.80174

BN—formulation based only on chloramphenicol (without the silica carriers); A_ClPh—formulation based on carrier A and chloramphenicol (1:1); D_ClPh—formulation based on carrier D and chloramphenicol (1:1).

**Table 7:** Values of the estimated matching parameters for profiles along with statistics, significance level, and confidence interval.

Sample	Parameter	Estimate	Standard Error	t-Statistic	*p*-Value	Confidence Interval (−)	Confidence Interval (+)
BN	k	0.00282	0.00016	18.115	1.82121 × 10^−6^	0.00244	0.00320
BN	b	0.54563	0.02018	27.033	1.69271 × 10^−7^	0.49624	0.59501
D_ClPh	k	0.00834	0.00042	20.077	9.91565 × 10^−7^	0.00732	0.00935
D_ClPh	b	0.55761	0.02187	25.496	2.39898 × 10^−7^	0.50409	0.61112
A_ClPh	k	0.006992	0.000185	37.8444	2.27263 × 10^−8^	0.00654	0.00654
A_ClPh	b	0.896888	0.025522	35.1416	3.53872 × 10^−8^	0.834438	0.834438

BN—formulation based only on chloramphenicol (without the silica carriers); A_ClPh—formulation based on carrier A and chloramphenicol (1:1); D_ClPh—formulation based on carrier D and chloramphenicol (1:1).

**Table 8:** Mahalanobis distance (MSD).

Comparison of the Profiles	Distance between the Profiles	Lower Limit of the Confidence Interval	Upper Limit of the Confidence Interval	Acceptable Distance between Profiles
BN vs. A_ClPh	13.1292	6.29682	19.9616	8.06196
BN vs. D_ClPh	16.502	9.670	23.334	9.709

BN—formulation based only on chloramphenicol (without the silica carriers); A_ClPh—formulation based on carrier A and chloramphenicol (1:1); D_ClPh—formulation based on carrier D and chloramphenicol (1:1).

## Data Availability

Data available within article and supplementary materials.

## Supplementary Materials

The following supporting information can be downloaded at: https://www.mdpi.com/article/10.3390/ph15060703/s1. Supplementary Figures S1–S29 and Tables S1–S37.
