# Supplementary material for: Nano-Silica Carriers Coated by Chloramphenicol: Synthesis, Characterization, and Grinding Trial as a Way to Improve the Release Profile
Source: Pharmaceuticals (Basel). 2022 Jun 2;15(6):703. doi: 10.3390/ph15060703 (PMC9230014; doi:10.3390/ph15060703)
Supplement: Supplementary file 1 [file pharmaceuticals-15-00703-s001.zip › pharmaceuticals-1732332-supplementary.pdf]

Supplementary Materials

Nano-silica carriers coated by chloramphenicol: synthesis, characterization, and grinding trial as a way to improve the re-lease profile

Radosław Balwierz [1*], Dawid Bursy [2], Paweł Biernat [2], Nataliia Hudz [1,3], Mariia Shanaida [4], Łukasz Krzemiński [5], Paweł Skóra [6], Monika Biernat [7] and Wioletta Ochędzan Siodłak [1]

[1] Faculty of Chemistry, University of Opole, Oleska 48, 45-052 Opole, Poland; radoslaw.balwierz@uni.opole.pl (R.B); wsiodlak@uni.opole.pl (W.S).

[2] Department of Drug Forms Technology, Faculty of Pharmacy, Wroclaw Medical University, 50-556 Wroclaw, Poland; dawid.bursy@umed.wroc.pl (D.B); pawel.biernat@umed.wroc.pl (P.B).

[3] Department of Drug Technology and Biopharmaceutics, Danylo Halytsky Lviv National Medical University, 79010 Lviv, Ukraine; natali_gudz@ukr.net

[4] Department of Pharmacognosy and Medical Botany, I. Horbachevsky Ternopil National Medical Uni-versity, 46001 Ternopil, Ukraine; shanayda@tdmu.edu.ua

[5] Nanotechnology and Materials Technology Scientific and Didactic Laboratory, Faculty of Mechanical Engineering, Silesian University of Technology, 44-100 Gliwice, Poland; lukasz.krzeminski@polsl.pl

[6] Department of Inorganic Chemistry, Analytical Chemistry and Electrochemistry, Faculty of Chemistry, Silesian University of Technology, 44-100 Gliwice, Poland; pawel.skora@polsl.pl

[7] Department of Haematology, Blood Neoplasms and Bone Marrow Transplantation, Wroclaw Medical University, 50-367 Wroclaw, Poland; mobiernat@gmail.com

* Correspondence: radoslaw.balwierz@uni.opole.pl

Table of contents	p.
**The scheme of the experiment**	4
**Section 1: Material analysis**	5
**Figure S1.** BET and BJH analysis of silica carrier **A**	5
**Figure S2.** BET and BJH analysis of silica carrier **B**	6
**Figure S3.** BET and BJH analysis of silica carrier **C**	7
**Figure S4.** BET and BJH analysis of silica carrier **D**	8
**Figure S5.** BET and BJH analysis of silica carrier **E**	9
**Figure S6.** BET and BJH analysis of silica carrier **F**	10
**Figure S7.** BET and BJH analysis silica carrier with chloramphenicol **D_ClPh**	11
**Figure S8.** Particle size distribution of silica carrier **A**	12
**Figure S9.** Particle size distribution of silica carrier **B**	12
**Figure S10.** Particle size distribution of silica carrier **C**	13
**Table S1.** Statistical summary of particle size measurements for silica carriers **A-C** obtained using the dynamic light scattering (DLS) method	13
**Figure S11.** Particle size distribution of silica carrier **D**	13
**Figure S12.** Particle size distribution of silica carrier **E**	14
**Figure S13.** Particle size distribution of silica carrier **F**	14
**Table S2.** Statistical summary of particle size measurements for silica carriers **D-F** obtained using the dynamic light scattering (DLS) method	14
**Figure S14.** Particle size distribution of silica carrier with chloramphenicol **A_ClPh**	15

Supplementary Materials

**Figure S15.** Particle size distribution of grinded silica carrier with chloramphenicol **D_ClPh**	15
**Table S3.** Statistical summary of particle size measurements for silica materials **A_ClPh** and **D_ClPh** obtained using the dynamic light scattering (DLS) method	15
**Figure S16.** XRD pattern of silica carrier **A**	16
**Figure S17.** XRD pattern of silica carrier with chloramphenicol **A_ClPh**	16
**Figure S18.** TGA curves of silica carrier **A**	17
**Figure S19.** TGA curves of silica carrier **B**	17
**Figure S20.** TGA curves of silica carrier **C**	18
**Figure S21.** TGA curves of grinded silica carrier **D**	18
**Figure S22.** TGA curves of grinded silica carrier **E**	19
**Figure S23.** TGA curves of silica carrier **F**	19
**Figure S24.** TGA curves of silica carrier with chloramphenicol **A_ClPh**	20
**Figure S25.** TGA curves of grinded silica carrier with chloramphenicol **D_ClPh**	20
**Section 2: Dissolution study of chloramphenicol from carbopol-based gels**	21
**Table S4.** Volume of acceptor fluid during release testing	21
**Figure S26.** Calibration curve for chloramphenicol at 278 nm	21
**Table S5.** Weights of pure chloramphenicol gel without silica nanoparticles (BN) and calculated total contents BN	22
**Table S6**. Weights chloramphenicol gel with grinded silica nanoparticles and calculated total contents **D_ClPh**	22
**Table S7.** Weights of chloramphenicol gel with silica nanoparticles and calculated total contents **A_ClPh**	22
**Table S8.** Calculated amount of chloramphenicol in the dissolution vessel after time, from formulation without silica nanoparticles BN.	22
**Table S9.** Calculated amount of chloramphenicol in the dissolution vessel after time, from formulation with grinded silica nanoparticles **D_ClPh**	23
**Table S10.** Calculated amount of chloramphenicol in the dissolution vessel after time, from formulation with silica nanoparticles **A_ClPh**	23
**Table S11.** Calculated amounts of chloramphenicol that left the vessel after a given time point from formulation without silica nanoparticles BN	23
**Table S12.** Calculated amounts of chloramphenicol that left the vessel after a given time point from formulation with grinded silica nanoparticles **D_ClPh**	24
**Table S13.** Calculated amounts of chloramphenicol that left the vessel after a given time point from formulation with silica nanoparticles **A_ClPh**	24
**Table S14.** The cumulative amount of the released chloramphenicol from formulation without silica nanoparticles BN	25
**Table S15.** The cumulative amount of the released chloramphenicol from formulation with grinded silica nanoparticles **D_ClPh**	25
**Table S16.** The cumulative amount of the released chloramphenicol from formulation with silica nanoparticles **A_ClPh**	25

Supplementary Materials

**Table S17.** Released percent of active substance after each time point for all tested formulations.	26
**Section 3: Model dependent analysis**	27
**Table S18.** First-order kinetics model calculation	27
**Table S19.** First-order kinetics model parameters	27
**Table S20. Calculation of zero order kinetics**	28
**Table S21. Zero-order kinetics parameters** | **Table S22.** Higuchi model calculation	28
**Table S22.** Higuchi model calculation	29
**Table S23.** Higuchi model parameters	29
**Table S24.** Hixson-Crowell model calculation	30
**Table S25.** Hixson-Crowell model parameters	30
**Table S26.** Korsmeyer-Peppas model calculation	31
**Table S27.** Korsmeyer-Peppas model Parameters	31
**Section 4: Model independent analysis**	32
**Table S28.** Analysis of Variance, variable: DE	32
**Table S29.** Levene Test of Homogeneity of Variances, variable: DE	32
**Table S30.** Breakdown descriptive statistics, variable: DE	33
**Figure S27.** Categorized box whisker plot of dissolution efficiency	33
**Table S31.** Fisher's Least significant difference test, variable: DE	34
**Figure S28.** Probability plot, variable: DE	34
**Table S32.** Calculation of the Mean Dissolution time for each formulation	35
**Table S33.** Mean dissolution time (MDT) for tested formulations	35
**Section 5: Weibull Model**	36
**Table S34.** Weibull function nonlinear estimation parameters	36
**Figure S29.** Plot of the fit to the Weibull model. In red is the function for the **D_ClPh** formulation, in yellow for **A_ClPh** and in blue for BN	37
**Table S35.** ANOVA for the Weibull model	37
**Table S36.** Mahalanoubis distance (MSD)	38
**Table S37.** Similarity and difference coefficients (F1,F2)	38

Supplementary Materials

The scheme of the experiment

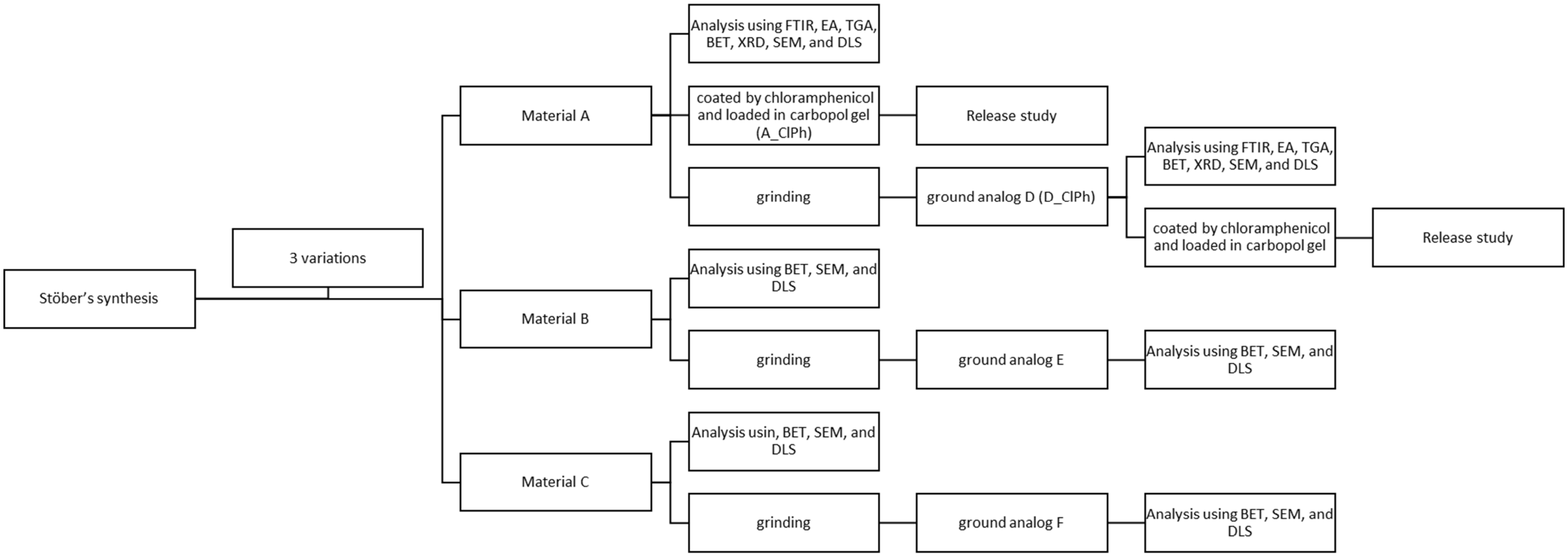

Materials A – C obtained using Stöber’s synthesis; material D – F obtained after grinding materials A – C; A_ClPh - formulation based on carrier A and chloramphenicol (1:1); D_ClPh - formulation based on carrier D and chloramphenicol (1:1); FTIR - Fourier transform infrared spectroscopy; , BET - Brunauer–Emmett–Teller method, EA - elemental analysis, TGA - thermogravimetric analysis, , XRD - X-ray diffraction study; SEM - scanning electron microscope; DLS - dynamic light scattering

Supplementary Materials

Section 1: Material analysis

Silica A

35,0244
±
0,1613
m²/g

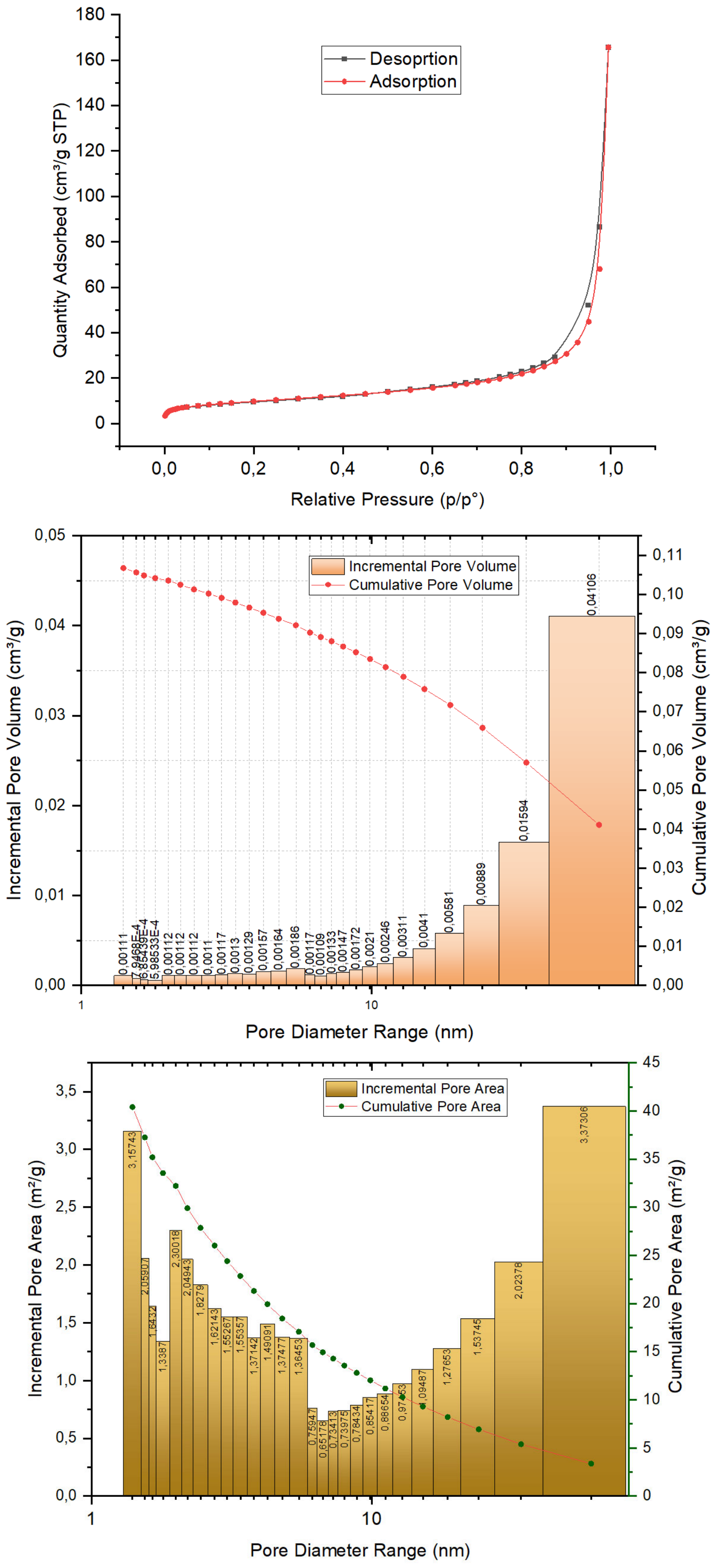

Figure S1. BET and BJH analysis of silica carrier **A**

Silica B

32,8918

±

0,3282 m²/g

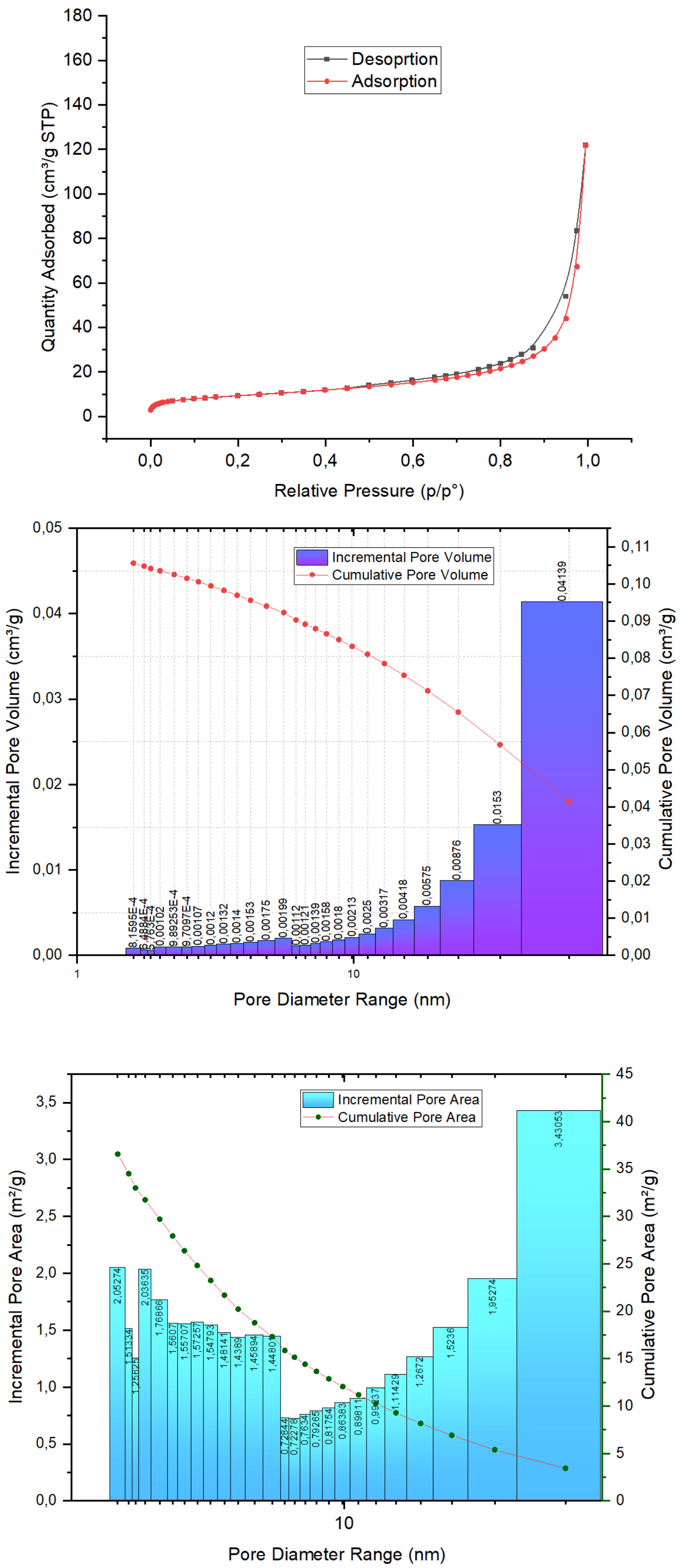

Figure S2. BET and BJH analysis of silica carrier **B**

Supplementary Materials

Silica C

32,7435
±
0,2081 m²/g

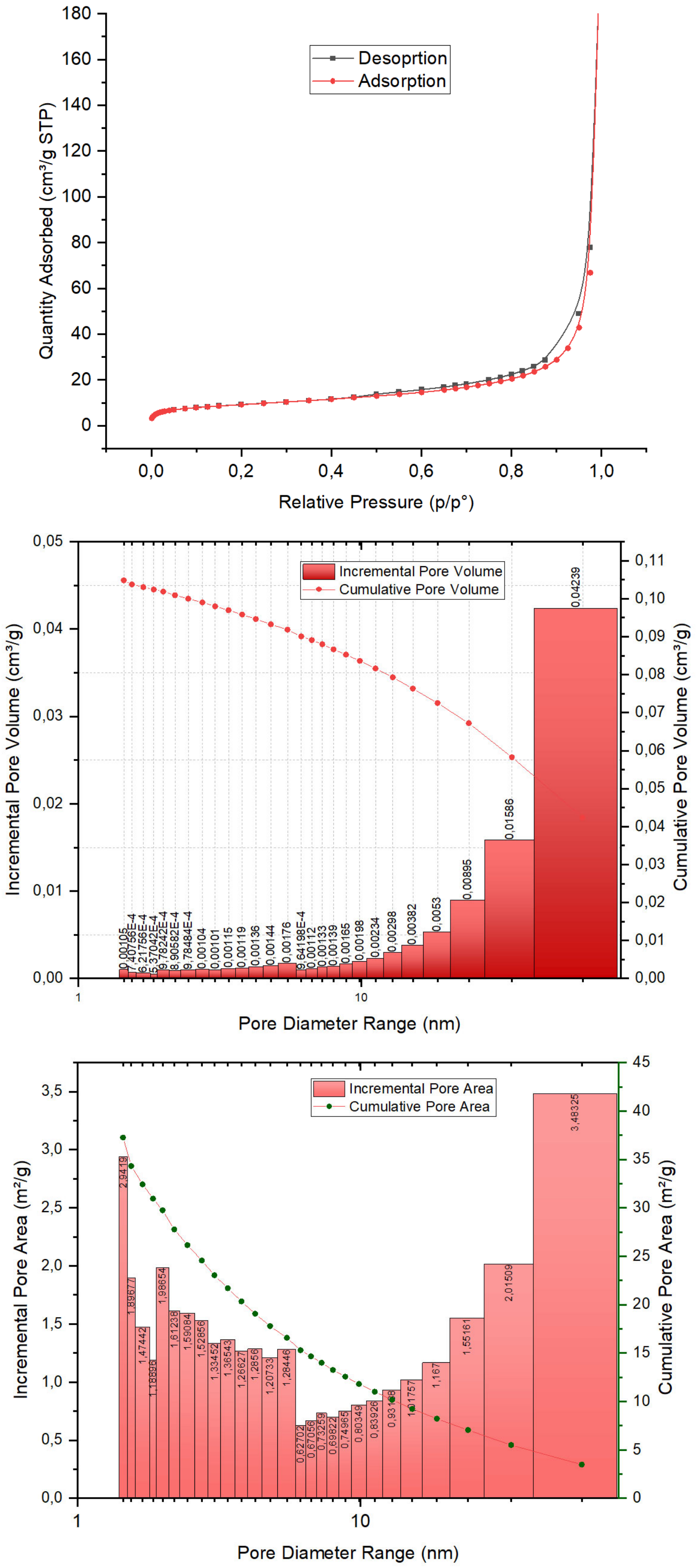

Figure S3. BET and BJH analysis of silica carrier **C**

Silica D

0,1871

±

0,0203 m²/g

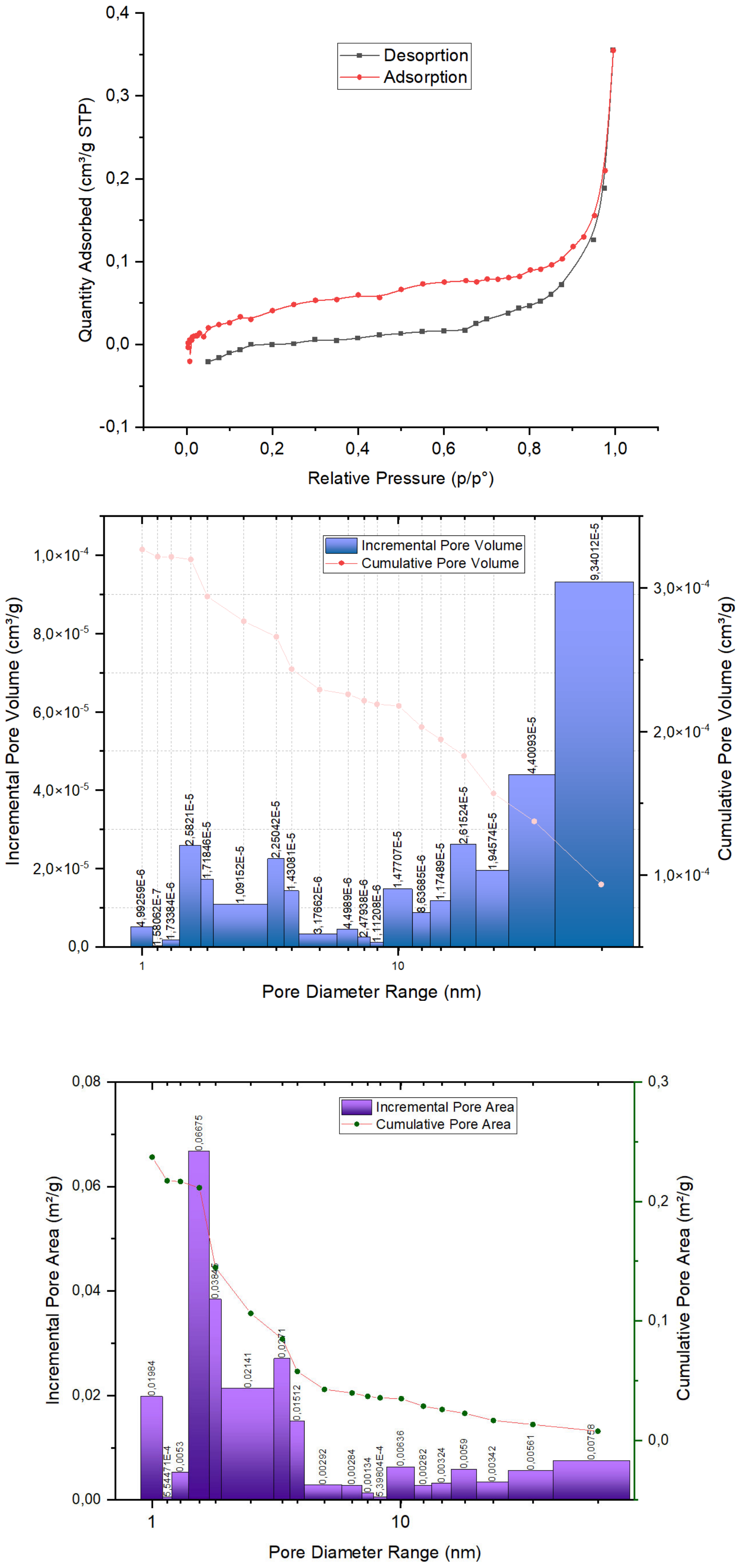

Figure S4. BET and BJH analysis of silica carrier **D**

Supplementary Materials

Silica E
13,8976 ±
0,0817 m²/g

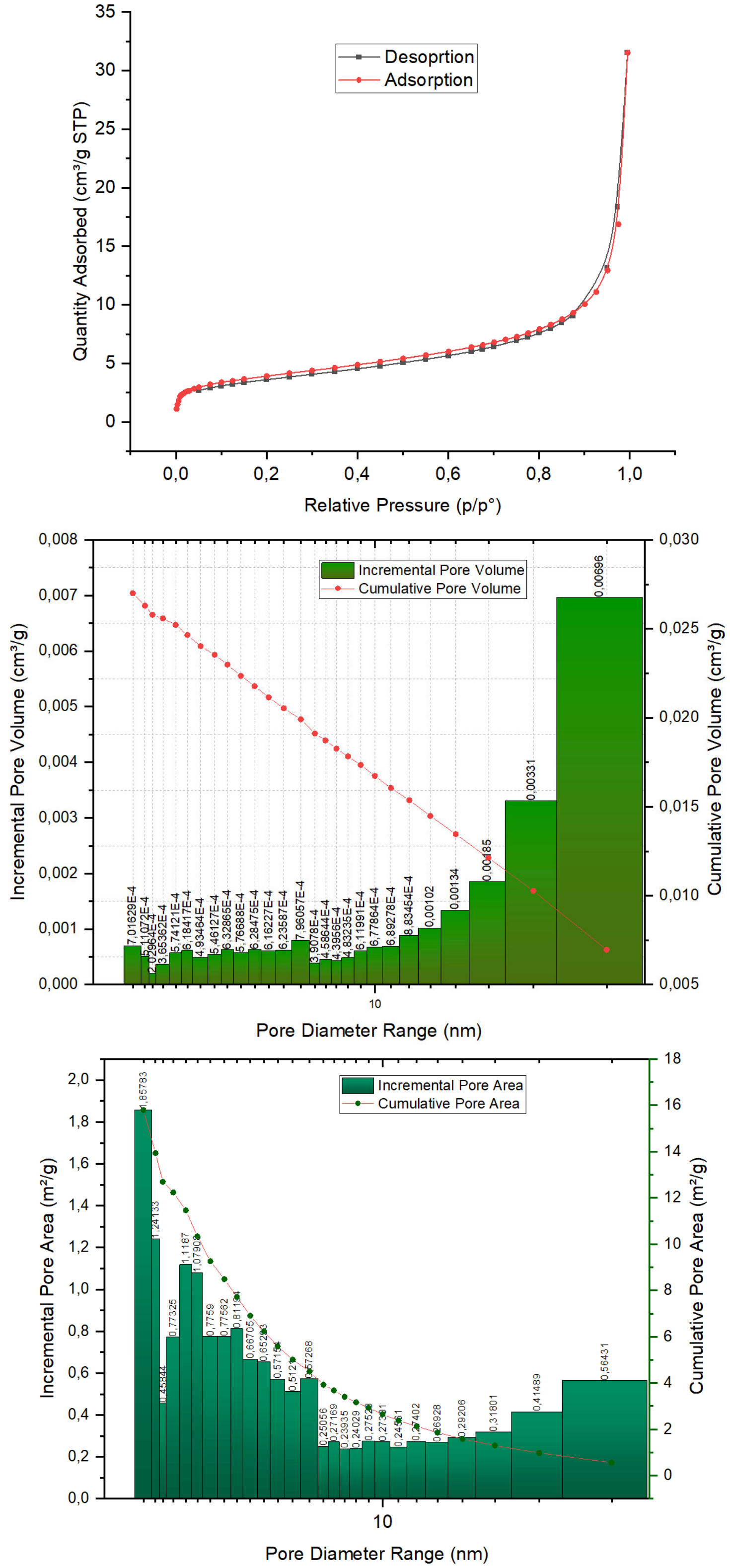

Figure S5. BET and BJH analysis of silica carrier **E**

Silica F

23,8035 ±
0,1482 m²/g

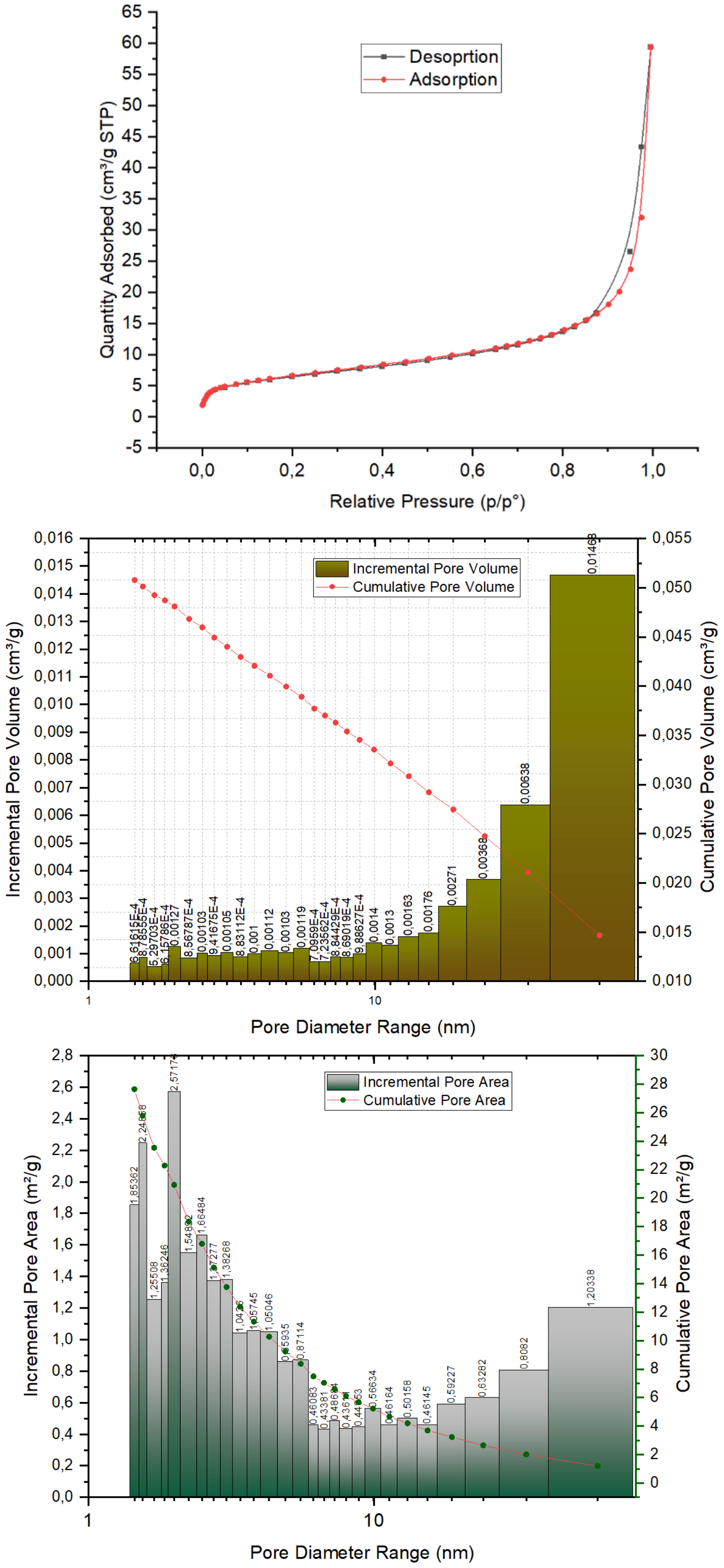

Figure S6. BET and BJH analysis of silica carrier **F**

Supplementary Materials

MATERIAL

D_ClPh

3,3736

±

0,0457 m²/g

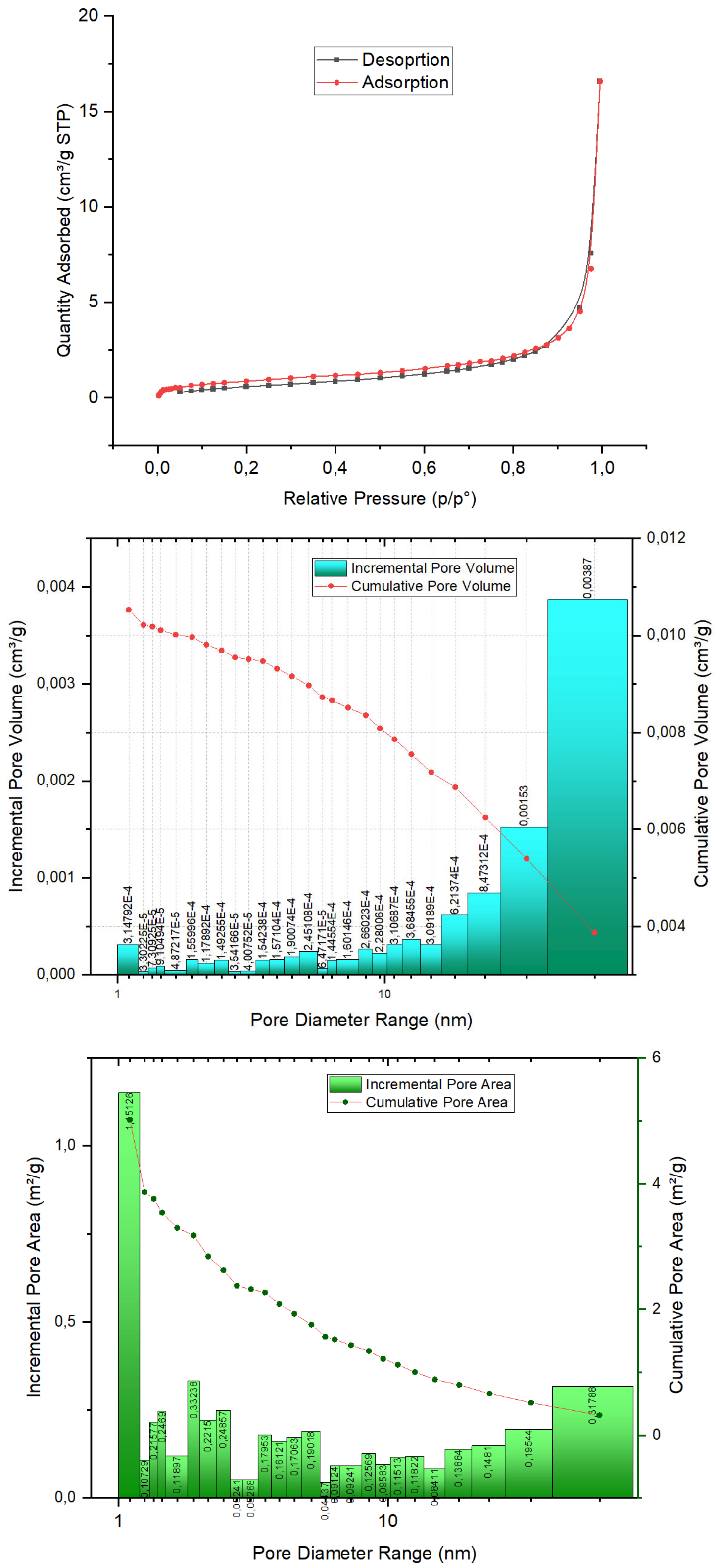

Figure S7. BET and BJH analysis of silica carrier with chloramphenicol **D_ClPh**

Supplementary Materials

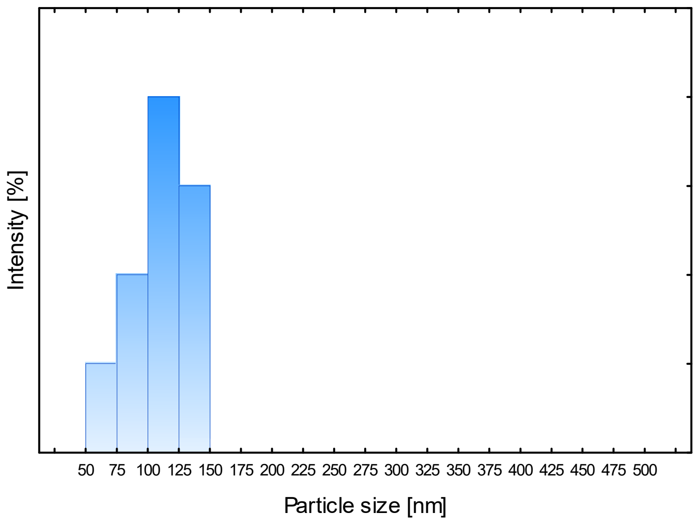

Figure S8. Particle size distribution of silica carrier **A**

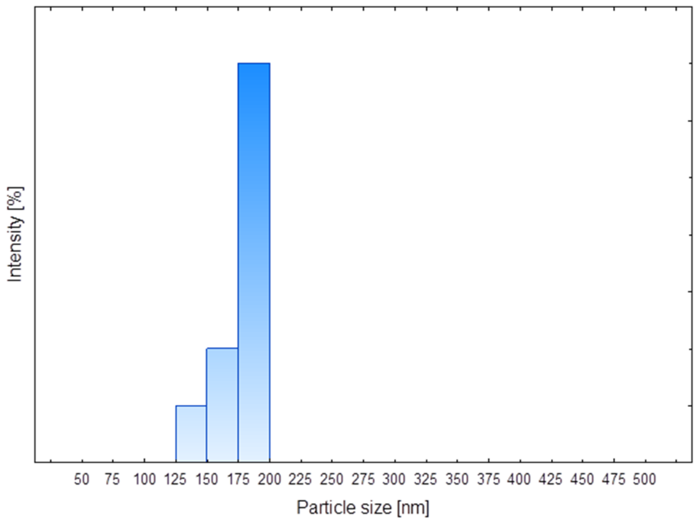

Figure S9. Particle size distribution of silica carrier **B**

Supplementary Materials

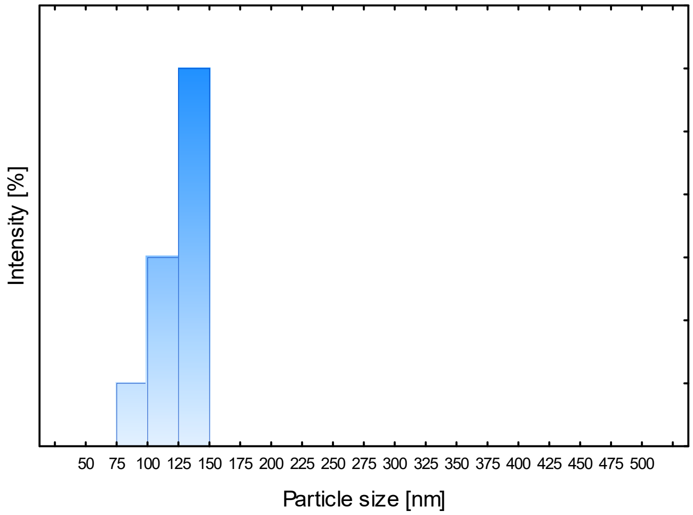

Figure S10. Particle size distribution of silica carrier **C**

Table S1. Statistical summary of particle size measurements for silica carriers **A-C** obtained using the dynamic light scattering (DLS) method

Silica carrier	Mean	Std.Dev.	RSD	Minimum	Median	Maximum
**A**	107.7	22.0	20.4%	62.8	112.8	131.0
**B**	181.4	16.0	8.8%	143.8	186.6	196.9
**C**	125.1	16.0	12.8%	84.2	131.9	137.3

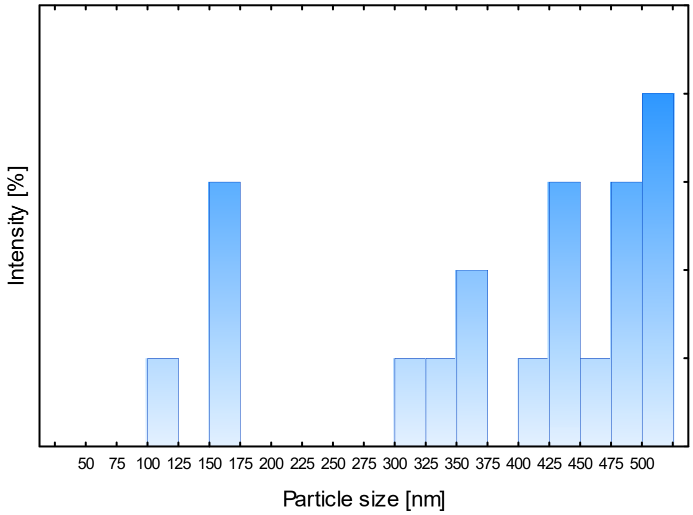

Figure S11. Particle size distribution of silica carrier **D**

Supplementary Materials

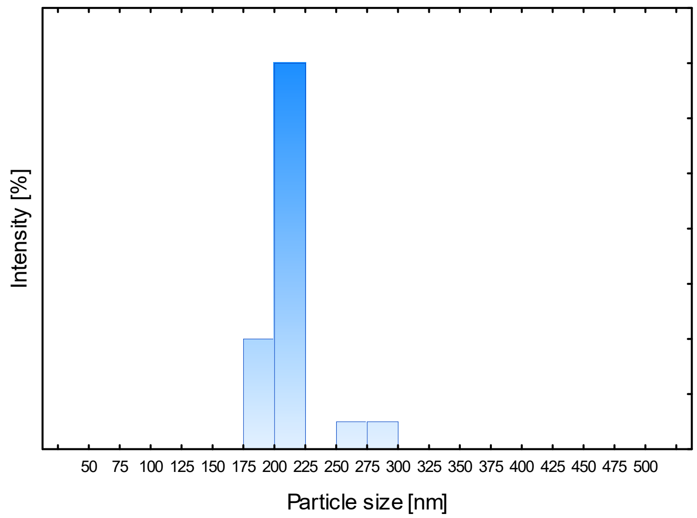

Figure S12. Particle size distribution of silica carrier **E**

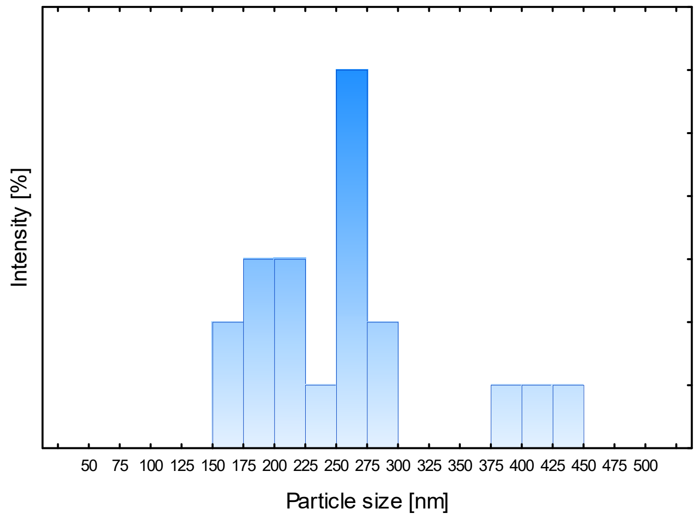

Figure S13. Particle size distribution of silica carrier **F**

Table S2. Statistical summary of particle size measurements for silica carriers **D-F** obtained using the dynamic light scattering (DLS) method

Grinded silica carrier	Mean	Std.Dev.	RSD	Minimum	Median	Maximum
**D**	389.7	139.3	35.7%	112.2	438.0	556.5
**E**	215.3	23.3	10.8%	188.8	212.9	289.2
**F**	260.0	77.8	29.9%	168.7	257.3	440.4

Supplementary Materials

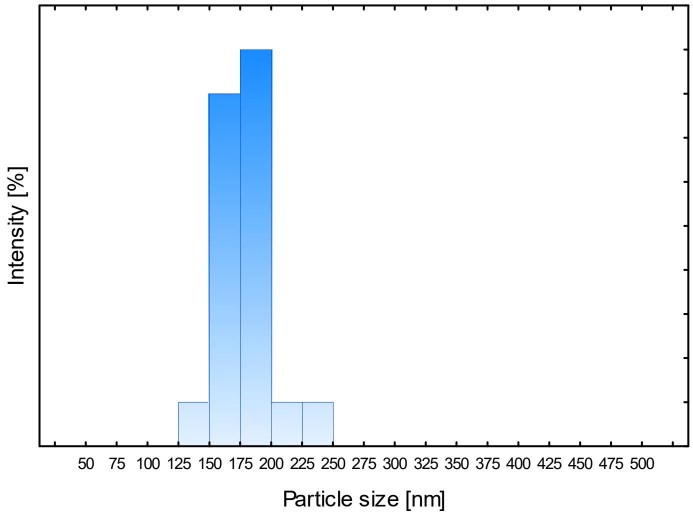

Figure S14. Particle size distribution of silica carrier with chloramphenicol **A_ClPh**

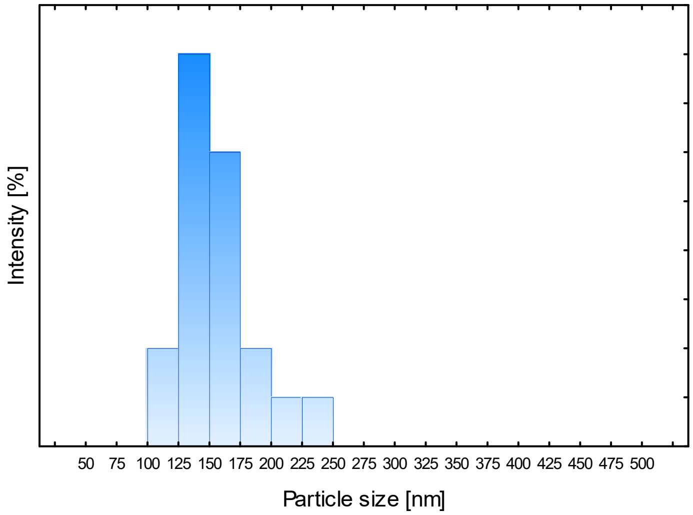

Figure S15. Particle size distribution of grinded silica carrier with chloramphenicol **D_ClPh**

Table S3. Statistical summary of particle size measurements for silica materials **A_ClPh** and **D_ClPh** obtained using the dynamic light scattering (DLS) method

Material	Mean	Std.Dev.	RSD	Minimum	Median	Maximum
**A_ClPh**	178.2	22.14	12.0%	141.8	182.2	241.2
**D_ClPh**	156.3	32.3	20.7%	100.3	151.8	229.1

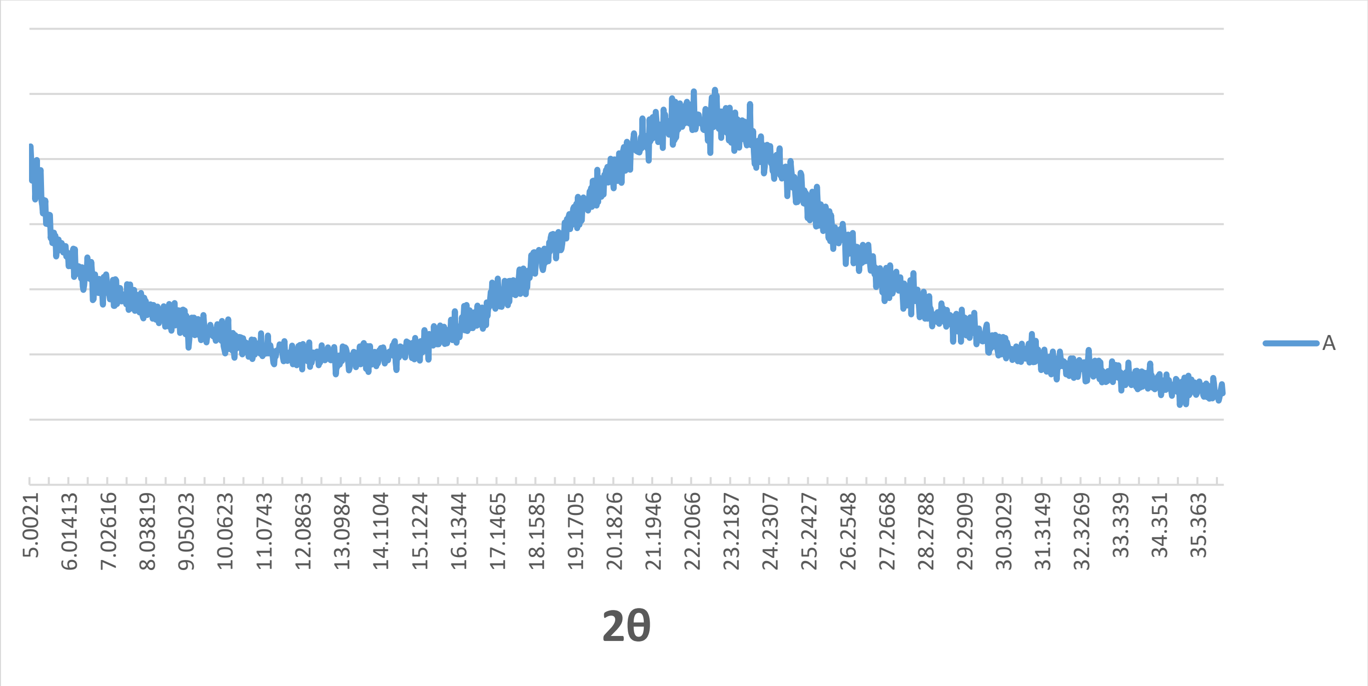

Figure S16. XRD pattern of silica carrier **A**

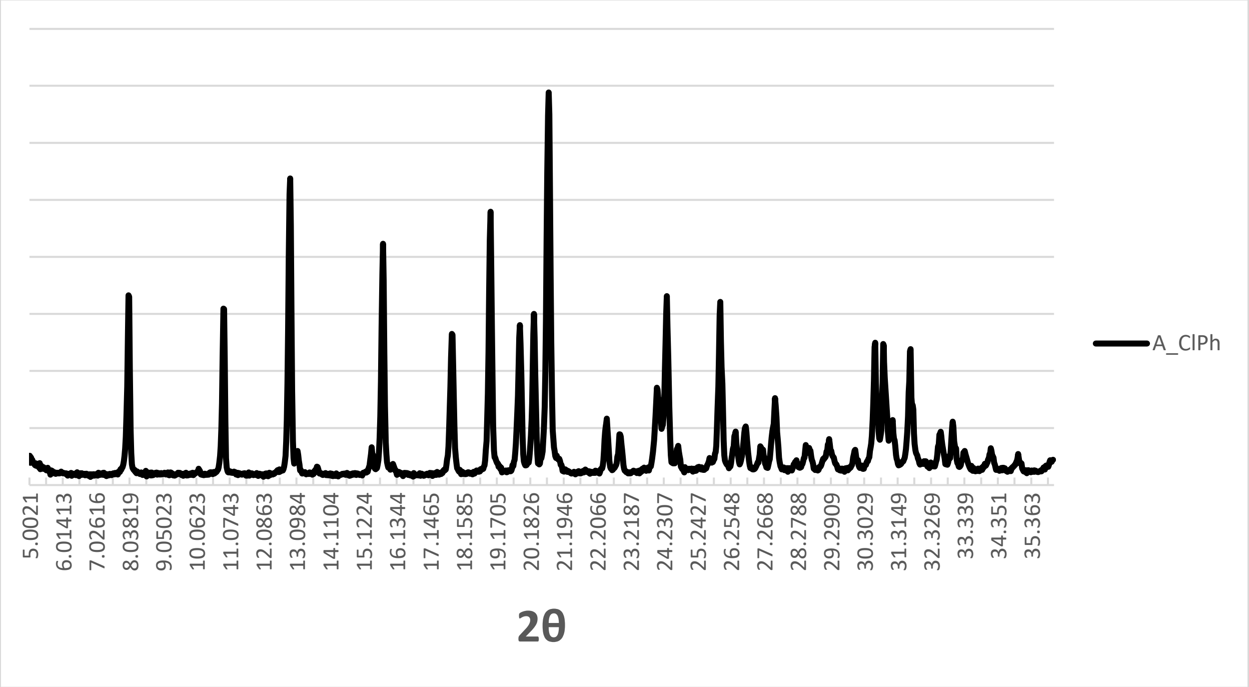

Figure S17. XRD pattern of silica carrier with chloramphenicol **A_ClPh**

Supplementary Materials

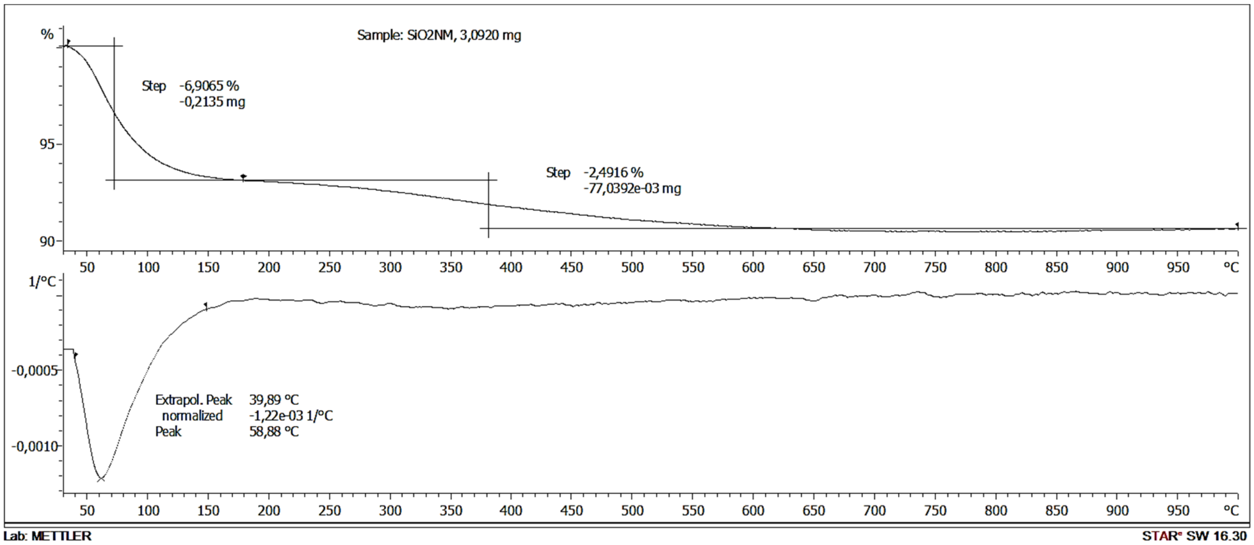

Figure S18. TGA curves of silica carrier **A**

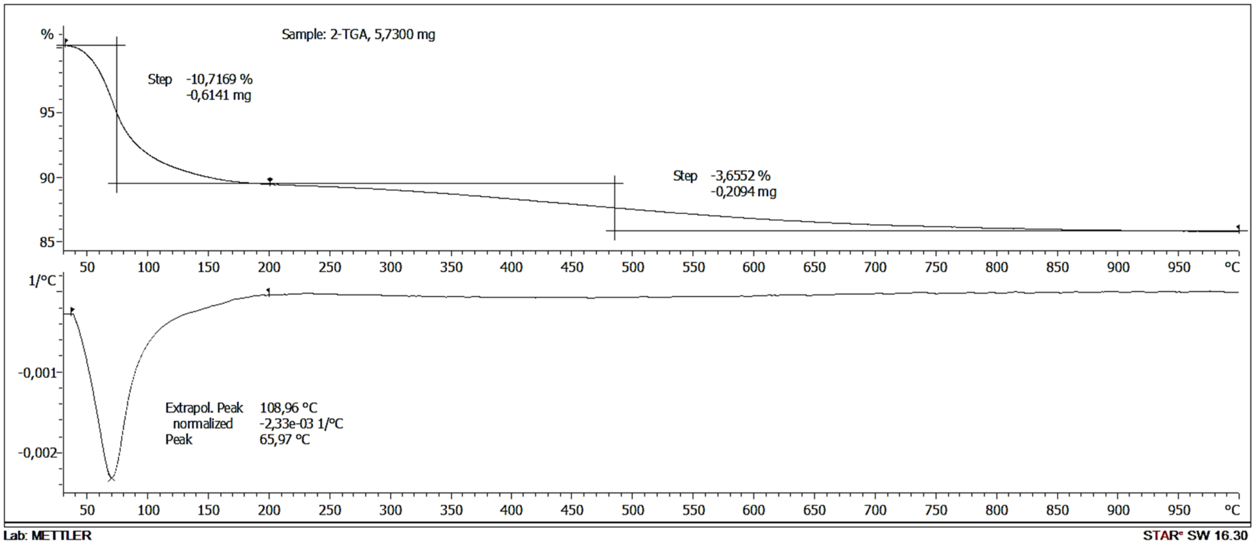

Figure S19. TGA curves of silica carrier **B**

Supplementary Materials

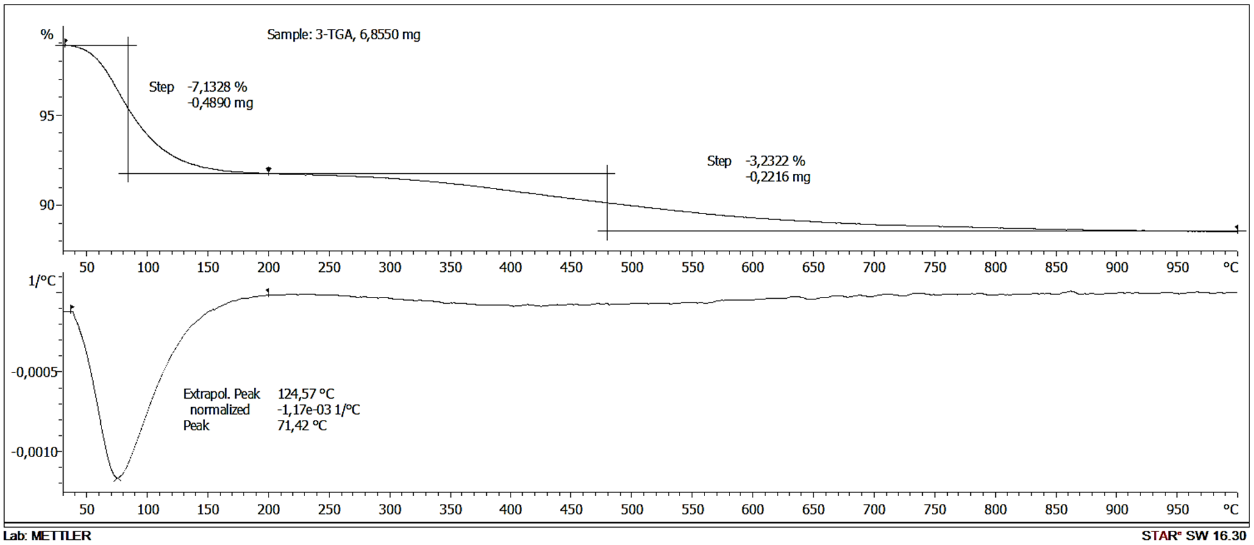

Figure S20. TGA curves of silica carrier **C**

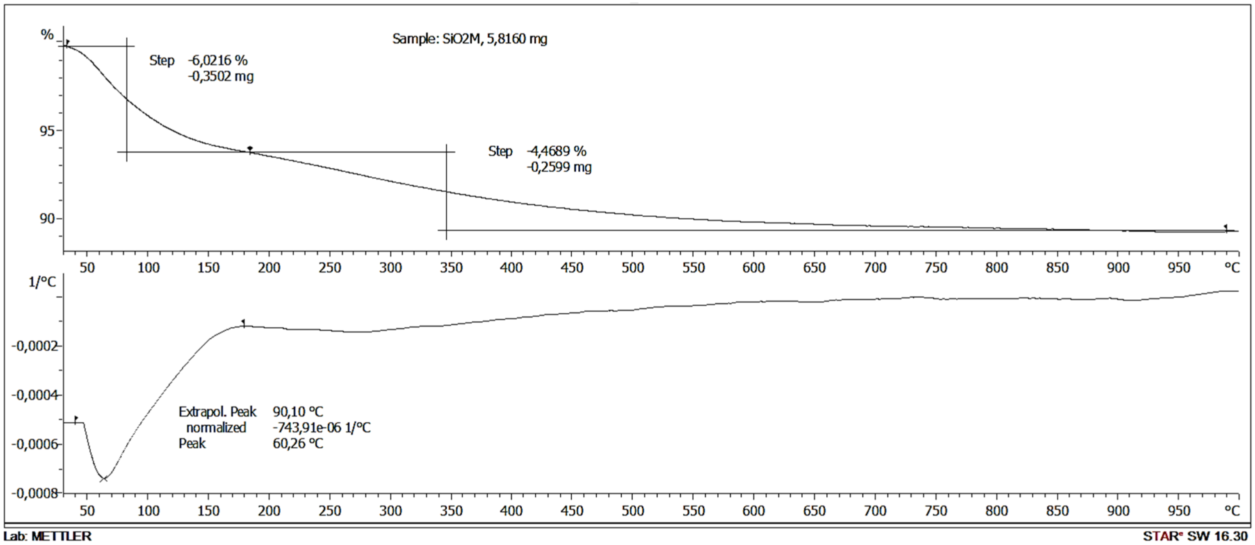

Figure S21. TGA curves of grinded silica carrier **D**

Supplementary Materials

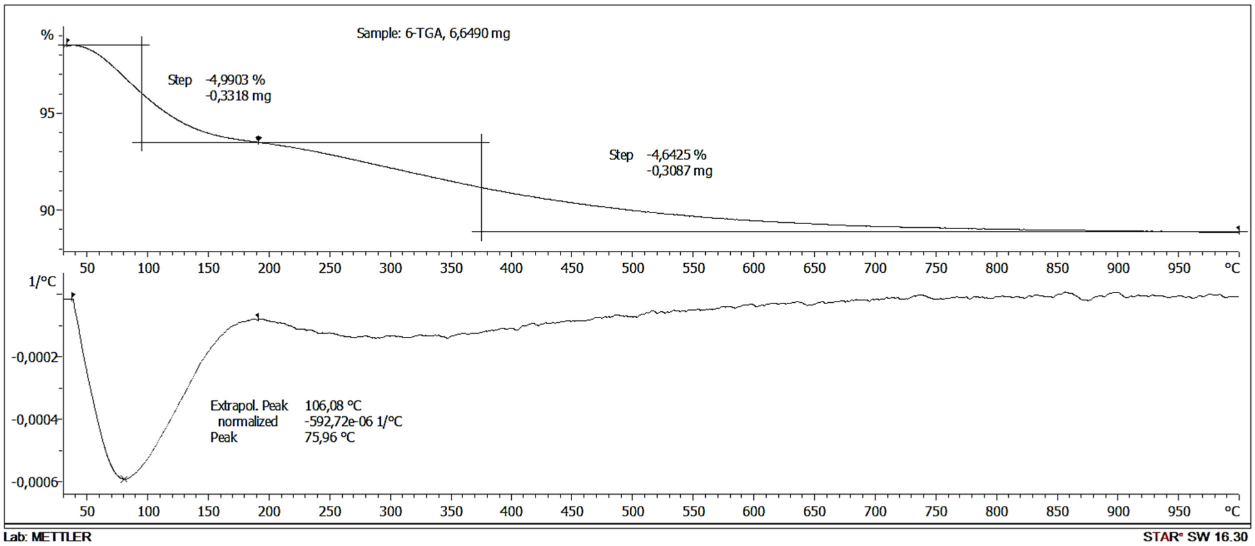

Figure S22. TGA curves of grinded silica carrier **E**

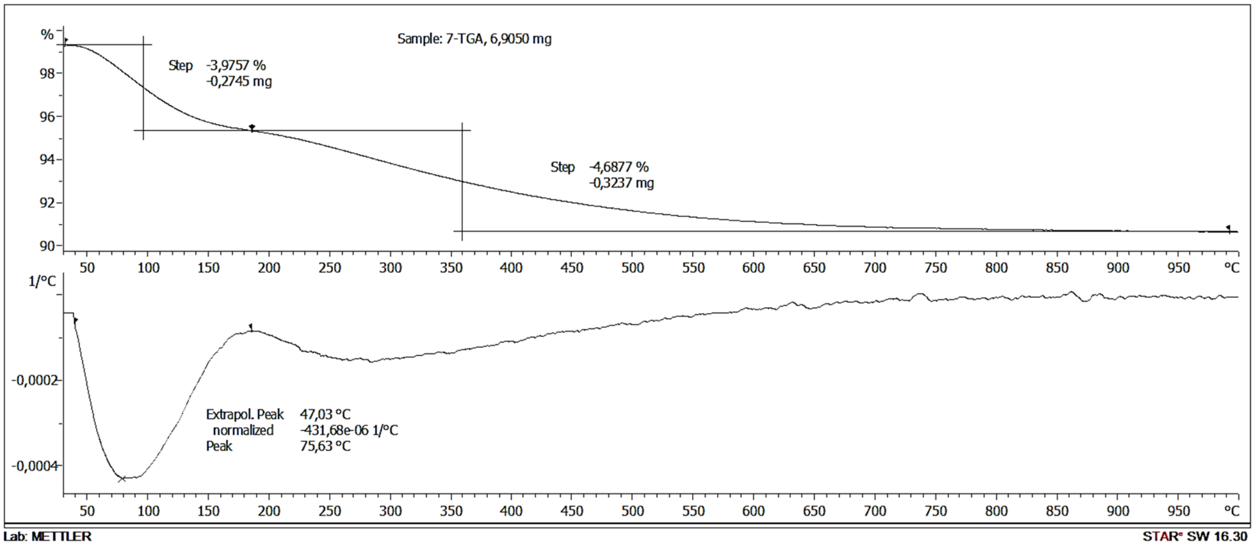

Figure S23. TGA curves of silica carrier **F**

Supplementary Materials

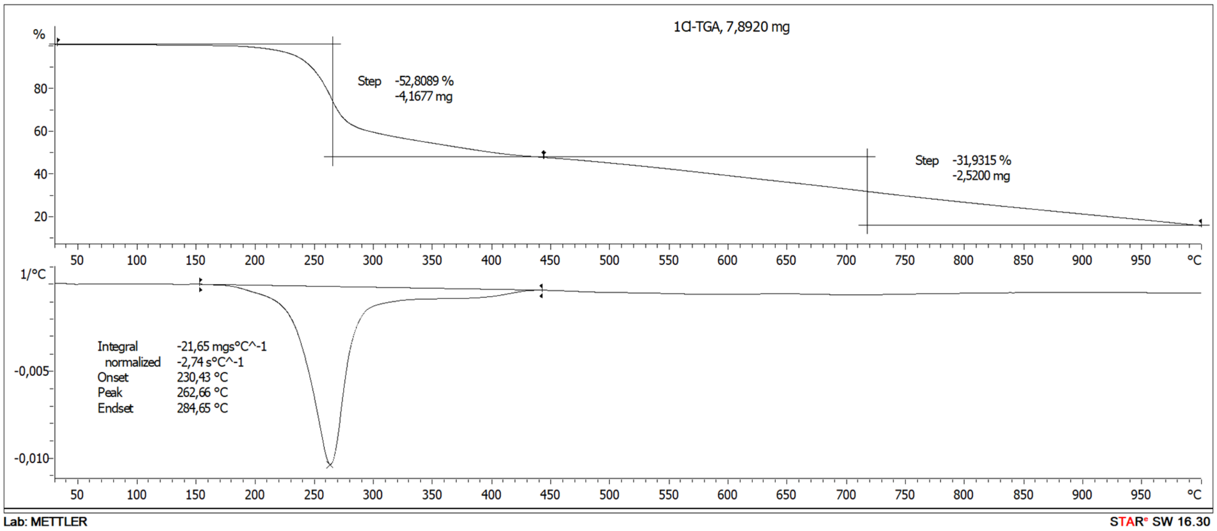

Figure S24. TGA curves of silica carrier with chloramphenicol **A_ClPh**

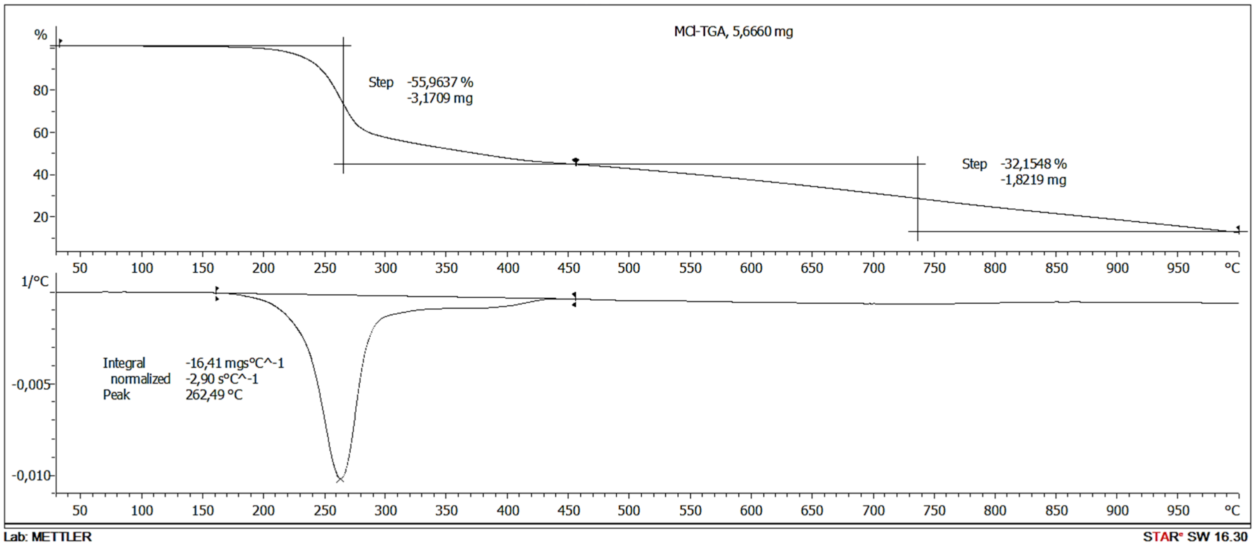

Figure S25. TGA curves of grinded silica carrier with chloramphenicol **D_ClPh**

Supplementary Materials

Section 2: Dissolution study of chloramphenicol from carbopol-based gels

The study was performed using immersion chambers according to the USP1724 monograph. The initial volume of acceptor fluid was 100 ml. At selected time points, the autosampler sampled a solution volume of 2 ml from each chamber and 0.5 ml for rinsing the sampling needles. The medium was not replenished after subsequent time points and the loss of acceptor at subsequent time points was taken into account.

Table S4. Volume of acceptor fluid during release testing

No.	time [min.]	Volume [ml]
**1**	**5**	100
**2**	**10**	97.5
**3**	**15**	95
**4**	**30**	92.5
**5**	**60**	90
**6**	**120**	87.5
**7**	**240**	85
**8**	**360**	82.5

Samples of the acceptor solution collected by autosampler were analyzed spectrophotometrically at λ = 278 nm.

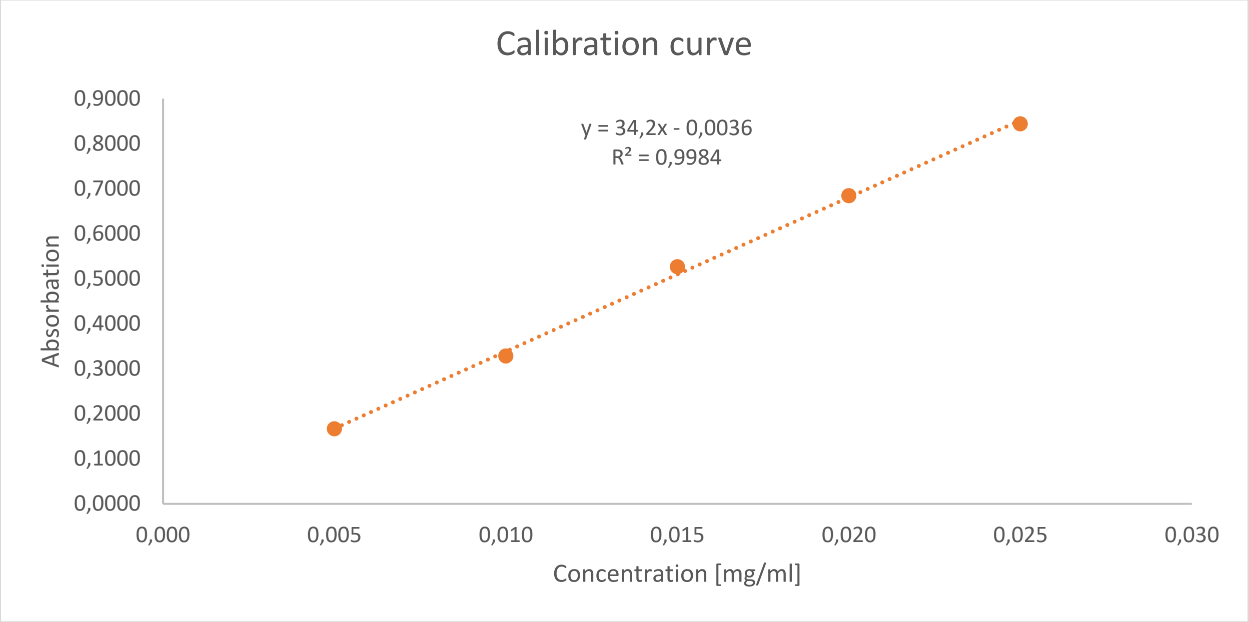

Figure S26. Calibration curve for chloramphenicol at 278 nm

The amount of active ingredient in the sample weight for each test unit was calculated based on the previously determined active ingredient content of the gel. The amount of released active substance in acceptor fluid Q1 was calculated for each unit (1-6) and each time point (1-8).

Then, the content of active substance in the solution taken for quantitative analysis was calculated Q2 after each timepiont.

Table S5. Weights of pure chloramphenicol gel without silica nanoparticles (BN) and calculated total contents BN.

Unit	1	2	3	4	5	6
**concentration [mg/100g]**			492.637			
**Weight of sample [g]**	0.672	0.648	0.682	0.680	0.646	0.644
**amount of active ingredient [mg]**	3.311	3.192	3.360	3.350	3.182	3.173

Table S6. Weights chloramphenicol gel with grinded silica nanoparticles and calculated total contents **D_ClPh**.

Unit	1	2	3	4	5	6
**concentration [mg/100g]**			502.3665			
**Weight of sample [g]**	0.635	0.624	0.645	0.651	0.654	0.670
**amount of active ingredient [mg]**	3.190	3.285	3.240	3.270	3.135	3.366

Table S7. Weights of chloramphenicol gel with silica nanoparticles and calculated total contents **A_ClPh**.

Unit	1	2	3	4	5	6
**concentration [mg/100g]**			528.857			
**Weight of sample [g]**	0.591	0.570	0.560	0.545	0.574	0.550
**amount of active ingredient [mg]**	3.126	3.014	2.962	2.882	3.036	2.909

Table S8. Calculated amount of chloramphenicol in the dissolution vessel after time, from formulation without silica nanoparticles BN.

No. of time point	Q1_unit 1 [mg]	Q1_unit 2 [mg]	Q1_unit 3 [mg]	Q1_unit 4 [mg]	Q1_unit 5 [mg]	Q1_unit 6 [mg]
1	0.1863	0.2243	0.7641	0.6042	0.3641	0.3091
2	0.3074	0.2969	0.5990	0.5449	0.4865	0.4001
3	0.4098	0.3822	0.7054	0.5904	0.4573	0.4626
4	0.6157	0.5863	0.7690	0.7853	0.6520	0.6640
5	0.9185	0.8560	1.0066	0.9938	0.9908	0.9027
6	1.2222	1.2249	1.2841	1.3570	1.3224	1.3314
7	1.6989	1.6530	1.5016	1.7238	1.7472	1.7063
8	1.9629	1.8789	1.9670	1.9904	1.9879	1.9286

Table S9. Calculated amount of chloramphenicol in the dissolution vessel after time, from formulation with grinded silica nanoparticles **D_ClPh**.

No. of time point	Q1_unit 1 [mg]	Q1_unit 2 [mg]	Q1_unit 3 [mg]	Q1_unit 4 [mg]	Q1_unit 5 [mg]	Q1_unit 6 [mg]
1	0.6625	0.7027	0.5888	0.4746	0.3388	0.4619
2	0.7510	0.8127	0.7555	0.6400	0.4886	0.8581
3	0.8012	0.9452	0.8763	0.9496	0.9077	0.9012
4	1.1028	1.0865	1.1102	1.3118	1.2193	1.1859
5	1.3769	1.2955	1.4491	1.4424	1.5002	1.5202
6	1.8105	1.8007	1.8121	1.9058	1.8753	1.8210
7	2.3399	2.3532	2.2703	2.4789	2.3128	2.3496
8	2.4745	2.5575	2.4470	2.6787	2.5866	2.6963

Table S10. Calculated amount of chloramphenicol in the dissolution vessel after time, from formulation with silica nanoparticles **A_ClPh**.

No. of time point	Q1_unit 1 [mg]	Q1_unit 2 [mg]	Q1_unit 3 [mg]	Q1_unit 4 [mg]	Q1_unit 5 [mg]	Q1_unit 6 [mg]
1	0.0873	0.1023	0.1314	0.1526	0.1906	0.1379
2	0.1951	0.2825	0.2595	0.3202	0.2382	0.3462
3	0.3204	0.3883	0.3578	0.4741	0.4458	0.3396
4	0.6378	0.6555	0.6315	0.7134	0.7387	0.5942
5	0.9659	1.0774	1.0497	1.0884	1.1485	1.0149
6	1.5026	1.5990	1.6115	1.5912	1.6477	1.5601
7	2.1629	2.2114	2.2411	2.1978	2.3986	2.2803
8	2.5459	2.5067	2.6441	2.4838	2.7290	2.5820

Table S11. Calculated amounts of chloramphenicol that left the vessel after a given time point from formulation without silica nanoparticles BN.

No. of time point	Σ Q2_unit 1 [mg]	Σ Q2_unit 2 [mg]	Σ Q2_unit 3 [mg]	Σ Q2_unit 4 [mg]	Σ Q2_unit 5 [mg]	Σ Q2_unit 6 [mg]
1	0.0047	0.0056	0.0191	0.0151	0.0091	0.0077
2	0.0125	0.0132	0.0345	0.0291	0.0216	0.0180
3	0.0233	0.0233	0.0530	0.0446	0.0336	0.0302
4	0.0400	0.0391	0.0738	0.0658	0.0512	0.0481
5	0.0655	0.0629	0.1018	0.0934	0.0788	0.0732
6	0.1004	0.0979	0.1385	0.1322	0.1165	0.1112
7	0.1504	0.1465	0.1826	0.1829	0.1679	0.1614

Table S12. Calculated amounts of chloramphenicol that left the vessel after a given time point from formulation with grinded silica nanoparticles **D_ClPh**.

No. of time point	Σ Q2_unit 1 [mg]	Σ Q2_unit 2 [mg]	Σ Q2_unit 3 [mg]	Σ Q2_unit 4 [mg]	Σ Q2_unit 5 [mg]	Σ Q2_unit 6 [mg]
1	0.0166	0.0176	0.0147	0.0119	0.0085	0.0115
2	0.0358	0.0384	0.0341	0.0283	0.0210	0.0336
3	0.0569	0.0633	0.0572	0.0533	0.0449	0.0573
4	0.0867	0.0926	0.0872	0.0887	0.0778	0.0893
5	0.1250	0.1286	0.1274	0.1288	0.1195	0.1315
6	0.1767	0.1801	0.1792	0.1832	0.1731	0.1836
7	0.2455	0.2493	0.2460	0.2561	0.2411	0.2527

Table S13. Calculated amounts of chloramphenicol that left the vessel after a given time point from formulation with silica nanoparticles **A_ClPh**.

No. of time point	Σ Q2_unit 1 [mg]	Σ Q2_unit 2 [mg]	Σ Q2_unit 3 [mg]	Σ Q2_unit 4 [mg]	Σ Q2_unit 5 [mg]	Σ Q2_unit 6 [mg]
1	0.0022	0.0026	0.0033	0.0038	0.0048	0.0034
2	0.0072	0.0098	0.0099	0.0120	0.0109	0.0123
3	0.0156	0.0200	0.0194	0.0245	0.0226	0.0213
4	0.0329	0.0377	0.0364	0.0438	0.0426	0.0373
5	0.0597	0.0677	0.0656	0.0740	0.0745	0.0655
6	0.1026	0.1133	0.1116	0.1195	0.1215	0.1101
7	0.1662	0.1784	0.1775	0.1841	0.1921	0.1772

The cumulative value of the released active substance was then calculated according to the following formula.

$$Q_{i,n}\,[mg] = Qa_{i,n} + \sum_{1}^{i-1} Qb_{i,n}$$

Where *i* means the following time point and n means the vessel unit number.

Table S14. The cumulative amount of the released chloramphenicol from formulation without silica nanoparticles BN.

No. of time point	(Q1+ΣQ2)_ unit 1 [mg]	(Q1+ΣQ2)_ unit 2 [mg]	(Q1+ΣQ2)_ unit 3 [mg]	(Q1+ΣQ2)_ unit 4 [mg]	(Q1+ΣQ2)_ unit 5 [mg]	(Q1+ΣQ2)_ unit 6 [mg]
1	0.1863	0.2243	0.7641	0.6042	0.3641	0.3091
2	0.3121	0.3025	0.6181	0.5600	0.4956	0.4078
3	0.4223	0.3954	0.7398	0.6195	0.4789	0.4806
4	0.6390	0.6096	0.8221	0.8299	0.6856	0.6941
5	0.9585	0.8952	1.0804	1.0597	1.0420	0.9508
6	1.2877	1.2878	1.3859	1.4505	1.4012	1.4045
7	1.7993	1.7509	1.6401	1.8561	1.8637	1.8175
8	2.1133	2.0254	2.1496	2.1733	2.1558	2.0900

Table S15. The cumulative amount of the released chloramphenicol from formulation with grinded silica nanoparticles **D_ClPh**.

No. of time point	(Q1+ΣQ2)_ unit 1 [mg]	(Q1+ΣQ2)_ unit 2 [mg]	(Q1+ΣQ2)_ unit 3 [mg]	(Q1+ΣQ2)_ unit 4 [mg]	(Q1+ΣQ2)_ unit 5 [mg]	(Q1+ΣQ2)_ unit 6 [mg]
1	0.6625	0.7027	0.5888	0.4746	0.3388	0.4619
2	0.7676	0.8303	0.7702	0.6518	0.4971	0.8697
3	0.8371	0.9836	0.9104	0.9779	0.9287	0.9348
4	1.1597	1.1498	1.1674	1.3650	1.2642	1.2431
5	1.4636	1.3881	1.5363	1.5312	1.5781	1.6096
6	1.9354	1.9294	1.9395	2.0346	1.9948	1.9525
7	2.5166	2.5333	2.4495	2.6622	2.4859	2.5332
8	2.7200	2.8068	2.6930	2.9349	2.8277	2.9490

Table S16. The cumulative amount of the released chloramphenicol from formulation with silica nanoparticles **A_ClPh**.

No. of time point	(Q1+ΣQ2)_ unit 1 [mg]	(Q1+ΣQ2)_ unit 2 [mg]	(Q1+ΣQ2)_ unit 3 [mg]	(Q1+ΣQ2)_ unit 4 [mg]	(Q1+ΣQ2)_ unit 5 [mg]	(Q1+ΣQ2)_ unit 6 [mg]
1	0.0873	0.1023	0.1314	0.1526	0.1906	0.1379
2	0.1973	0.2851	0.2628	0.3240	0.2429	0.3496
3	0.3276	0.3981	0.3677	0.4861	0.4567	0.3519
4	0.6534	0.6756	0.6509	0.7379	0.7613	0.6154
5	0.9988	1.1151	1.0861	1.1322	1.1910	1.0522
6	1.5623	1.6666	1.6771	1.6652	1.7222	1.6256
7	2.2655	2.3248	2.3527	2.3172	2.5202	2.3904
8	2.7121	2.6851	2.8216	2.6679	2.9211	2.7592

Supplementary Materials

The percentage released active substance is calculated as ratio of the amount realeased and the total amount of active substance in each vessel unit. Mean value and the standard deviation of each time point is presented in the following table.

Table S17. Released percent of active substance after each time point for all tested formulations.

	Formulation	BN		D_ClPh		A_ClPh	
**No**	**time [min]**	**mean [%]**	**SD**	**mean [%]**	**SD**	**mean [%]**	**SD**
0	0	0	-	0	-	0	-
1	5	12.436	6.655	16.561	4.218	4.490	1.264
2	10	13.741	3.781	22.455	3.837	9.318	2.097
3	15	15.975	3.678	28.596	1.486	13.352	2.277
4	30	21.844	2.432	37.730	2.673	22.856	2.007
5	60	30.582	1.883	46.754	2.664	36.719	2.682
6	120	42.014	2.186	60.518	2.124	55.382	2.775
7	240	54.879	3.367	77.926	2.371	79.107	3.856
8	360	64.960	1.618	86.894	2.782	92.462	3.787

Supplementary Materials

Section 3: Model dependent analysis

First-order kinetics model

According to the first-order kinetics model, we determine a straight line that is the time dependence of the logarithm of the percent residue of the drug substance. Percent residue was determined from the equation PP[%] = 100 – Q%, then the values were logarithmized. Process parameters were determined in the form of a constant release rate k calculated as the slope of the straight line of the logarithm of the percentage of residue versus time.

Table S18. First-order kinetics model calculation

No.	Time [min]	BN		D_ClPh		A_ClPh	
		PP [%]	ln (PP)	PP [%]	ln (PP)	PP [%]	ln (PP)
1	**5**	87.564	4.4724	83.439	4.4241	95.510	4.5592
2	**10**	86.259	4.4574	77.545	4.3509	90.682	4.5074
3	**15**	84.025	4.4311	71.404	4.2684	86.648	4.4618
4	**30**	78.156	4.3587	62.270	4.1315	77.144	4.3457
5	**60**	69.418	4.2401	53.246	3.9749	63.281	4.1476
6	**120**	57.986	4.0602	39.482	3.6758	44.618	3.7981
7	**240**	45.121	3.8094	22.074	3.0944	20.893	3.0394
8	**360**	35.040	3.5565	13.106	2.5731	7.538	2.0200

Table S19. First-order kinetics model parameters.

Patameter	BN	D_ClPh	A_ClPh
k [%/min] =	0.0026	0.0051	0.0069
$t_{50\%}$ [min] =	268.630	136.695	100.465
PP_0 [%] =	85.128	77.019	97.940
R^2 =	0.983	0.990	0.996

Supplementary Materials

Zero order kinetics Model

According to zero-order kinetics, there is a linear dependence of both the percent released and the percent residual on time.

Table S20. Calculation of zero order kinetics

No.	Time [min]	BN PP [%]	D_ClPh PP [%]	A_ClPh PP [%]
1	**5**	87.564	83.439	95.510
2	**10**	86.259	77.545	90.682
3	**15**	84.025	71.404	86.648
4	**30**	78.156	62.270	77.144
5	**60**	69.418	53.246	63.281
6	**120**	57.986	39.482	44.618
7	**240**	45.121	22.074	20.893
8	**360**	35.040	13.106	7.538

Table S21. Zero-order kinetics parameters

Parameter	BN	D_ClPh	A_ClPh
k_{0_T} [%/min] =	0.14886	0.18849	0.2468
$PP_0\ T$ [%] =	83.576	72.612	86.705
R^2 =	0.940	0.897	0.932

Supplementary Materials

Higuchi's model

Higuchi's model is primarily used to describe the release of well and poorly soluble substances from matrix therapeutic systems, and is also used to describe the rate of penetration of an active substance through a membrane or skin. This model uses the dependence of the percent released on the square root of time.

Table S22. Higuchi model calculation.

No	time [min]	$t^{0.5}$ [$min^{0.5}$]	BN Q [%]	D_ClPh Q [%]	A_ClPh Q [%]
1	5	2.236	12.436	16.561	4.490
2	10	3.162	13.741	22.455	9.318
3	15	3.873	15.975	28.596	13.352
4	30	5.477	21.844	37.730	22.856
5	60	7.746	30.582	46.754	36.719
6	120	10.954	42.014	60.518	55.382
7	240	15.492	54.879	77.926	79.107
8	360	18.974	64.960	86.894	92.462

Table S23. Higuchi model parameters

Parameter	BN	D_ClPh	A_ClPh
a **=**	3.24798	4.18411	5.4066
b **=**	4.481	11.659	-6.688
R^2 **=**	0.997	0.985	0.996

Supplementary Materials

Hixson-Crowell model

Hixson-Crowell cube root law -describes the release from systems where there is a change in surface area and diameter of particles. To study the release kinetics, data obtained from in vitro drug release studies were plotted as cube root of drug percentage remaining in matrix versus time.

Table S24. Hixson-Crowell model calculation

Lp	**Time [min]**	$(PP_T)^{1/3}$	$(PP_T)^{1/3}$	$(PP_R)^{1/3}$
1	**5**	4.441	4.370	4.571
2	**10**	4.418	4.264	4.493
3	**15**	4.380	4.149	4.425
4	**30**	4.276	3.964	4.257
5	**60**	4.110	3.762	3.985
6	**120**	3.871	3.405	3.547
7	**240**	3.560	2.805	2.754
8	**360**	3.272	2.358	1.961

Table S25. Hixson-Crowell model parameters

**Parameter**	**BN**	**D_ClPh**	**A_ClPh**
k_{r_T} =	0.0033	0.0055	0.0072
R^2 =	0.971	0.970	0.994

Supplementary Materials

Korsmeyer-Peppas model

Korsmeyer-Peppas model is a simple relationship which described drug release from a polymeric system equation. To study the release kinetics, data obtained from in vitro drug release studies were plotted as log cumulative percentage drug release versus log time.

Table S26. Korsmeyer-Peppas model calculation

No.	time [min]	ln (t)	BN ln (Qt_{SR})	D_ClPh ln (Qt_{SR})	A_ClPh ln (Qt_{SR})
1	5	1.609	2.5206	2.8071	1.5018
2	10	2.303	2.6204	3.1115	2.2319
3	15	2.708	2.7711	3.3533	2.5917
4	30	3.401	3.0839	3.6304	3.1292
5	60	4.094	3.4204	3.8449	3.6033
6	120	4.787	3.7380	4.1029	4.0143
7	240	5.481	4.0051	4.3558	4.3708
8	360	5.886	4.1738	4.4647	4.5268

Table S27. Korsmeyer-Peppas model Parameters

***n*=**	0.4128	0.3838	0.6932
**ln (*a*) =**	1.730	2.256	0.623
**a=**	5.640	9.549	1.865
**R^2 =**	0.990	0.993	0.982

Supplementary Materials

Section 4: Model independent analysis

D.E. release efficiency

Based on the conducted pharmaceutical availability studies for each of the six units of the drug formulation belonging to the three compared formulations, the D.E. release efficiency was determined - a parameter independent of the adopted kinetic model. Basic descriptive statistics were first calculated for the three mean values of the parameter: deviation (SD) and standard error (SE), coefficient of variation (CV, RSD) and 95% significance interval for the mean (95%CI). The normality of their distributions was assessed by the Shapiro-Wilk W test. Levene's test was used to assess homogeneity of variance. All analyzed variables met the assumptions of both normality of distribution and homogeneity of variance. In the next stage the statistical significance of differences between the mean values of compared parameters was assessed using parametric analysis of variance AVOVA. For post-hoc multiple comparisons Fisher's NIR test of least significant difference was used. In all applied analyses and statistical tests, the significance level of α=0.05 was assumed.

Table S28. Analysis of Variance, variable: DE

SS Effect	df Effect	MS Effect	SS Error	df Error	MS Error	F	p
0.132840	2	0.066420	0.007693	15	0.000513	129.5104	0.000000

Marked effects are significant at p < .05000, variable: DE

Table S29. Levene Test of Homogeneity of Variances, variable: DE

SS Effect	df Effect	MS Effect	SS Error	df Error	MS Error	F	p
0.000173	2	0.000086	0.001855	15	0.000124	0.699202	0.512475

Marked effects are significant at p < .05000, variable: DE

Supplementary Materials

Table S30. Breakdown descriptive statistics, variable: DE

Parameter for variable: DE	Formulation			All Groups
	BN	D_ClPh	A_ClPh	
**Mean**	0.4562	0.6513	0.6221	0.5765
**Number**	6	6	6	18
**Std. Dev.**	0.0182	0.0184	0.0295	0.0909
**Std. Error**	0.0074	0.0075	0.0120	0.0214
**CV**	3.98%	2.83%	4.74%	15.77%
**Minimum**	0.4387	0.6356	0.5684	0.4387
**Maximum**	0.4805	0.6741	0.6498	0.6741
**Confidence level -0.95**	0.4371	0.6319	0.5911	0.5313
**Confidence level +0.95**	0.4752	0.6706	0.6530	0.6217

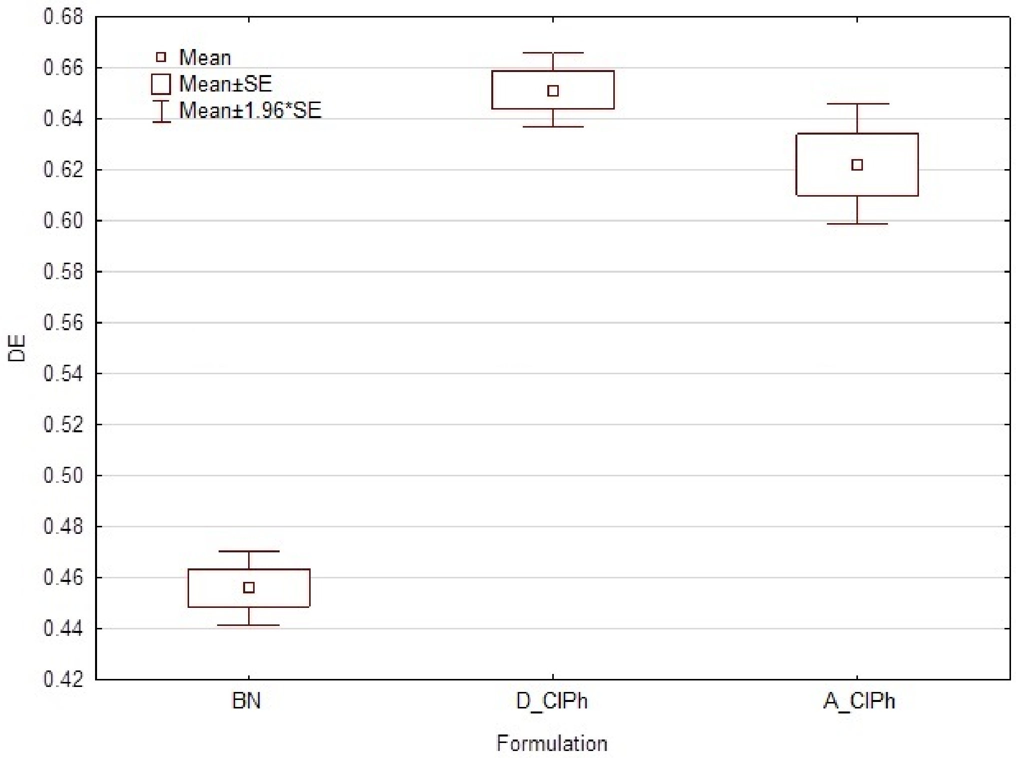

Figure S27. Categorized box whisker plot of dissolution efficiency.

Table S31. Fisher's Least significant difference test, variable: DE

LSD Test; Variable: DE

Marked differences are significant at p < .05000

Formulation	{1} M=,45618	{2} M=,65126	{3} M=,54027
**BN {1}**	X	2.09E-10	2.01E-09
**D_ClPh {2}**	2.09E-10	X	4.12E-02
**A_ClPh {3}**	2.01E-09	4.12E-02	X

Marked differences are significant at p < .05000, variable: DE

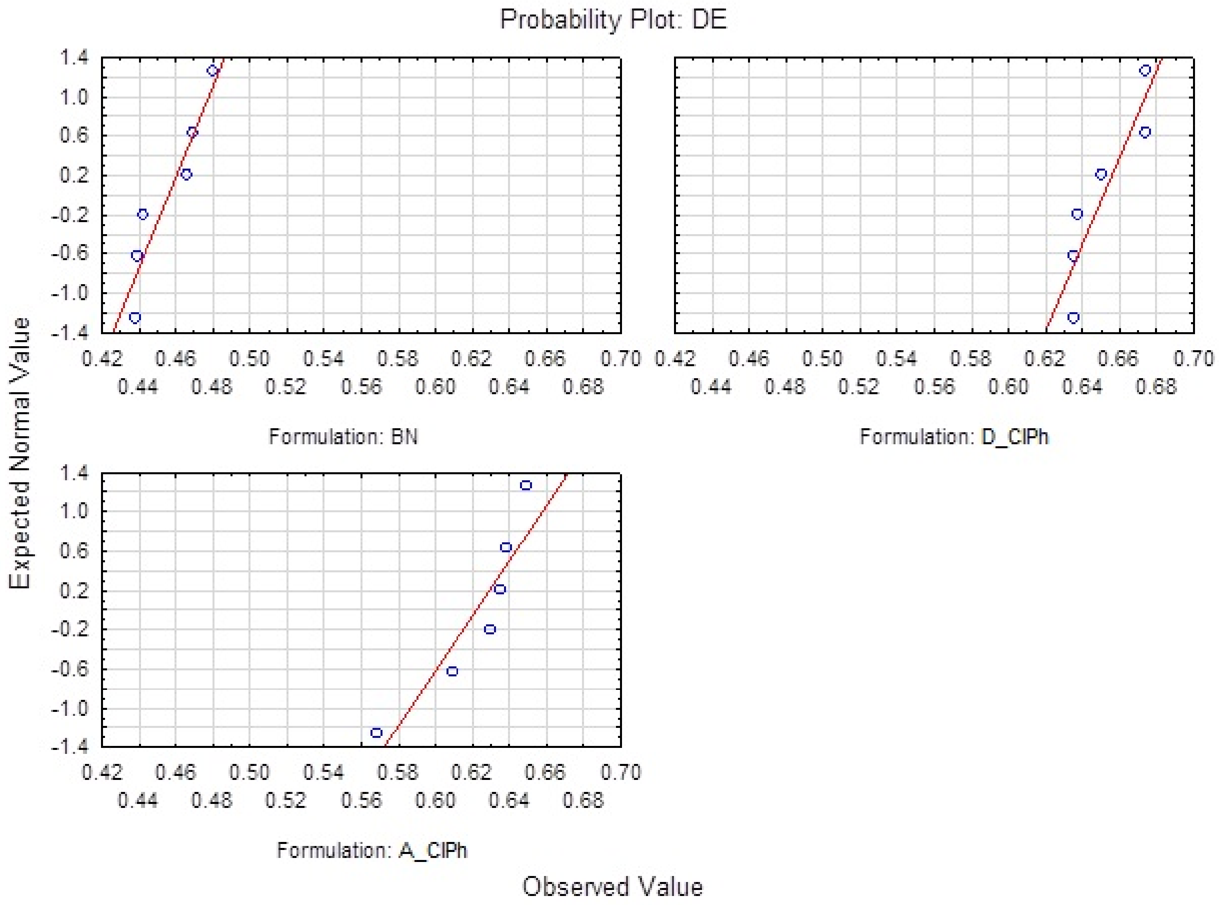

Figure S28. Probability plot, variable: DE

Supplementary Materials

MDT (Mean Dissolution Time)

Mean Dissolution Time MDT (Mean Dissolution Time) is equivalent to the first statistical moment MRT (Mean Drug Residence Time, Mean Residence Time). It is determined according to the following equation:

$$MDT = \frac{\sum_{i=1}^{n} \hat{t}_i \cdot \Delta M_i}{\sum_{i=1}^{n} \Delta M_i}, \quad \hat{t}_i = \frac{t_i + t_{i-1}}{2},$$

From the averaged value of the percent released and timepoints, the mean dissolution time (MDT) was calculated for each formulation as model-independent analysis. The analysis consists of determining the average time values between following time points including the zero point. The increment of the percent released relative to the previous one is then calculated. For each of the time points, the average time value is multiplied by the increment of the percent released. The results of the multiplication are summed and this value is divided by the value of the percent released at the last time point.

Table S32. Calculation of the Mean Dissolution time for each formulation

ti*	BN		D_ClPh		A_ClPh	
	**ΔMi**	**ti* • ΔMi**	**ΔMi**	**ti* • ΔMi**	**ΔMi**	**ti* • ΔMi**
2.5	12.436	31.089	16.561	41.404	4.490	11.224
7.5	1.305	9.787	5.894	44.202	4.828	36.211
12.5	2.235	27.933	6.141	76.761	4.035	50.434
22.5	5.868	132.034	9.134	205.508	9.503	213.828
45.0	8.739	393.245	9.024	406.093	13.863	623.824
90.0	11.432	1028.880	13.764	1238.786	18.663	1679.714
180.0	12.864	2315.544	17.408	3133.387	23.724	4270.398
300.0	10.081	3024.324	8.968	2690.499	13.355	4006.500
**∑ =**	***64.960***	***6962.837***	***86.894***	***7836.640***	***92.462***	***10892.132***

ti – time points; **ΔMi** averaged value of the percent released

Table S33. Mean dissolution time (MDT) for tested formulations.

Formulation	Mean dissolution time [min]
**BN**	107.187
**D_ClPh**	90.186
**A_ClPh**	117.802

Supplementary Materials

Section 5: Weibull Model

Nonlinear of release profiles were used model-dependent based on Weibull function.

The obtained three release profiles were described using the Weibull model recommended by FDA and EMA guidelines. The estimation of model parameters (k, b) was performed using the form of Weibull function defined according to the formula:

$$Qt = 100 * (1 - Exp(-k * t)^{b})$$

minimizing the S least squares loss function defined as:

$$S = \sum (Obs - Pred)^2$$

The minimization procedure for the loss function S so defined was performed based on a nonlinear Quasi-Newton iterative algorithm. The nonlinear estimation procedure was carried out using random initial values of the parameter estimators k and b. The initial step length in the iterative procedure was set as 0.001 by adopting a convergence criterion of 1*10^(-6).

The values of the estimated parameters in the fit are shown in the table below. Below is a graph showing the function run and the mean values of the percent released.

Table S34. Weibull function nonlinear estimation parameters

Sample	Parameter	Estimate	Standard Error	t-Statistic	P-Value	Confidence Interval(-)	Confidence Interval(+)
**BN**	k	0.00282	0.00016	18.115	1.82121 *10^-6	0.00244	0.00320
**BN**	b	0.54563	0.02018	27.033	1.69271 *10^-7	0.49624	0.59501
**D_ClPh**	k	0.00834	0.00042	20.077	9.91565 *10^-7	0.00732	0.00935
**D_ClPh**	b	0.55761	0.02187	25.496	2.39898 *10^-7	0.50409	0.61112
**A_ClPh**	k	0.006992	0.000185	37.8444	2.27263 *10^-8	0.00654	0.00654
**A_ClPh**	b	0.896888	0.025522	35.1416	3.53872 *10^-8	0.834438	0.834438

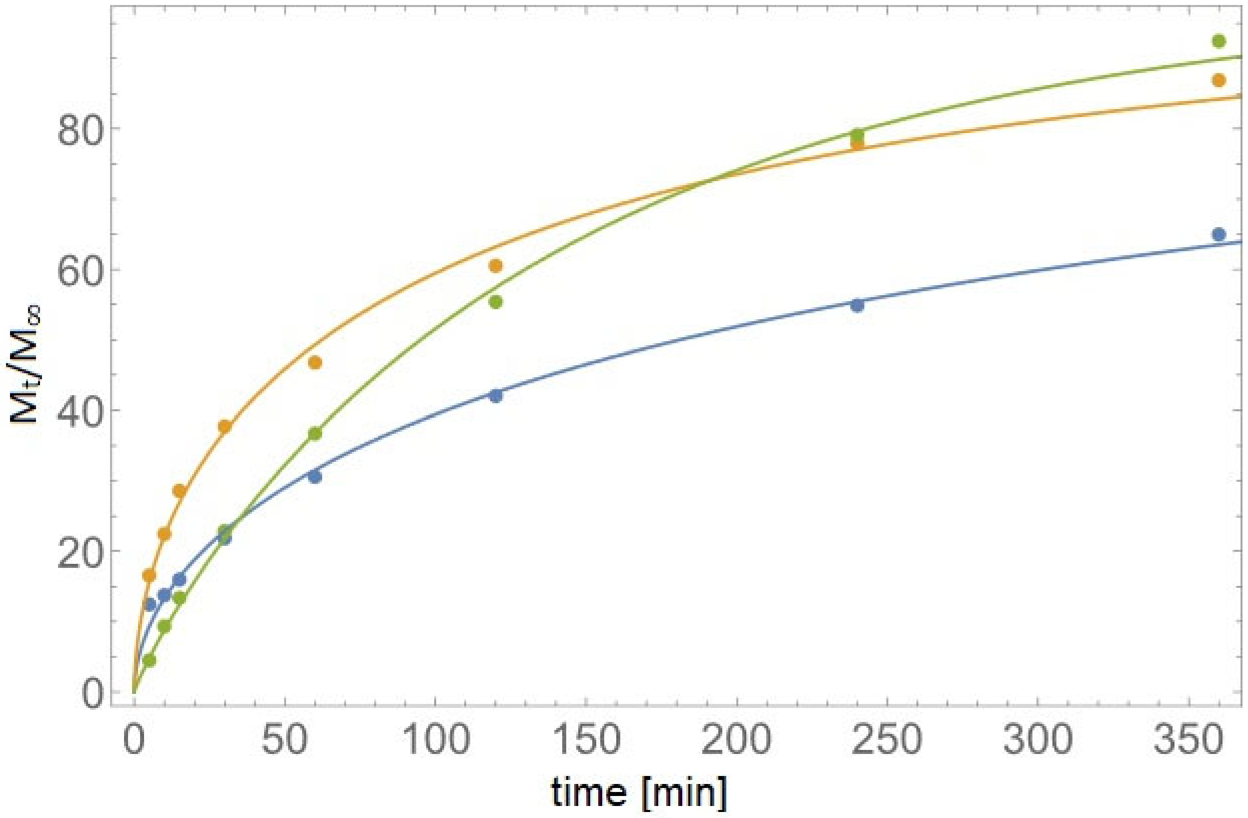

Figure S29. Plot of the fit to the Weibull model. In red is the function for the **D_ClPh** formulation, in yellow for **A_ClPh** and in blue for BN

Table S35. ANOVA for the Weibull model

	BN			D_ClPh			A_ClPh		
	**DF**	**SS**	**MS**	**DF**	**SS**	**MS**	**DF**	**SS**	**MS**
**Model**	2	10993	5496.48	2	22465	11232.5	2	20016.4	10008.2
**Error**	6	14.7527	2.45878	6	26.1519	4.35865	6	13.6398	2.27331
**Uncorrected Total**	8	11007.7		8	22491.2		8	20030.1	
**Corrected Total**	7	2788.13		7	4684.11		7	7730.33	

Supplementary Materials

The Mahalanobis distance

The Mahalanobis distance is the distance between release profiles that differentiates the respective components' contributions and exploits the correlations between them. We determined the mean, difference between the compared release profiles by determining the multivariate Mahalanoubis distance (MSD) for each pair (BN vs. A_ClPh and BN vs. D_ClPh) along with the recommended 90% significance interval

Table S36. Mahalanoubis distance (MSD)

Comparison of the profiles	Distance between the profiles	lower limit of the confidence interval	upper limit of the confidence interval	acceptable distance between profiles
**BN vs. A_ClPh**	13.1292	6.29682	19.9616	8.06196
**BN vs. D_ClPh**	16.502	9.670	23.334	9.709

Model independent coefficients of similarity and difference (F1,F2)

The parameters describing the release of 6 tested and reference formulation samples under identical conditions were determined, then for each time interval (j) the average percentage of released active substance from the tested formulation QT_j and the reference formulation QR_j was calculated. The release curves are considered similar when F1 is close to 0 (0-15) and F2 is close to 100 (50-100)

Table S37. Similarity and difference coefficients (F1,F2)

Formulation	Similarity factor F1 to the reference formulation (BN)	Difference factor F2 to reference formulation (BN)
**A_ClPh**	27.81	42.09
**D_ClPh**	47.19	39.37
